# Brew temperature, at fixed brew strength and extraction, has little impact on the sensory profile of drip brew coffee

**DOI:** 10.1038/s41598-020-73341-4

**Published:** 2020-10-05

**Authors:** Mackenzie E. Batali, William D. Ristenpart, Jean-Xavier Guinard

**Affiliations:** 1 Department of Food Science and Technology, University of California, Davis, One Shields Avenue, Davis, CA 95616 USA; 2 Department of Chemical Engineering, University of California, Davis, One Shields Avenue, Davis, CA 95616 USA

**Keywords:** Chemistry, Sustainability

## Abstract

The brew temperature is widely considered a key parameter affecting the final quality of coffee, with a temperature near 93 °C often described as optimal. In particular, drip brewers that do not achieve a minimum brew temperature of 92 °C within a prescribed time period fail their certification. There is little empirical evidence in terms of rigorous sensory descriptive analysis or consumer preference testing, however, to support any particular range of brew temperatures. In this study, we drip-brewed coffee to specific brew strengths, as measured by total dissolved solids (TDS), and extraction yields, as measured by percent extraction (PE), spanning the range of the classic Coffee Brewing Control Chart. Three separate brew temperatures of 87 °C, 90 °C, or 93 °C were tested, adjusting the grind size and overall brew time as necessary to achieve the target TDS and PE. Although the TDS and PE both significantly affected the sensory profile of the coffee, surprisingly the brew temperature had no appreciable impact. We conclude that brew temperature should be considered as only one of several parameters that affect the extraction dynamics, and that ultimately the sensory profile is governed by differences in TDS and PE rather than the brew temperature, at least over the range of temperatures tested.

## Introduction

**Figure 1:**
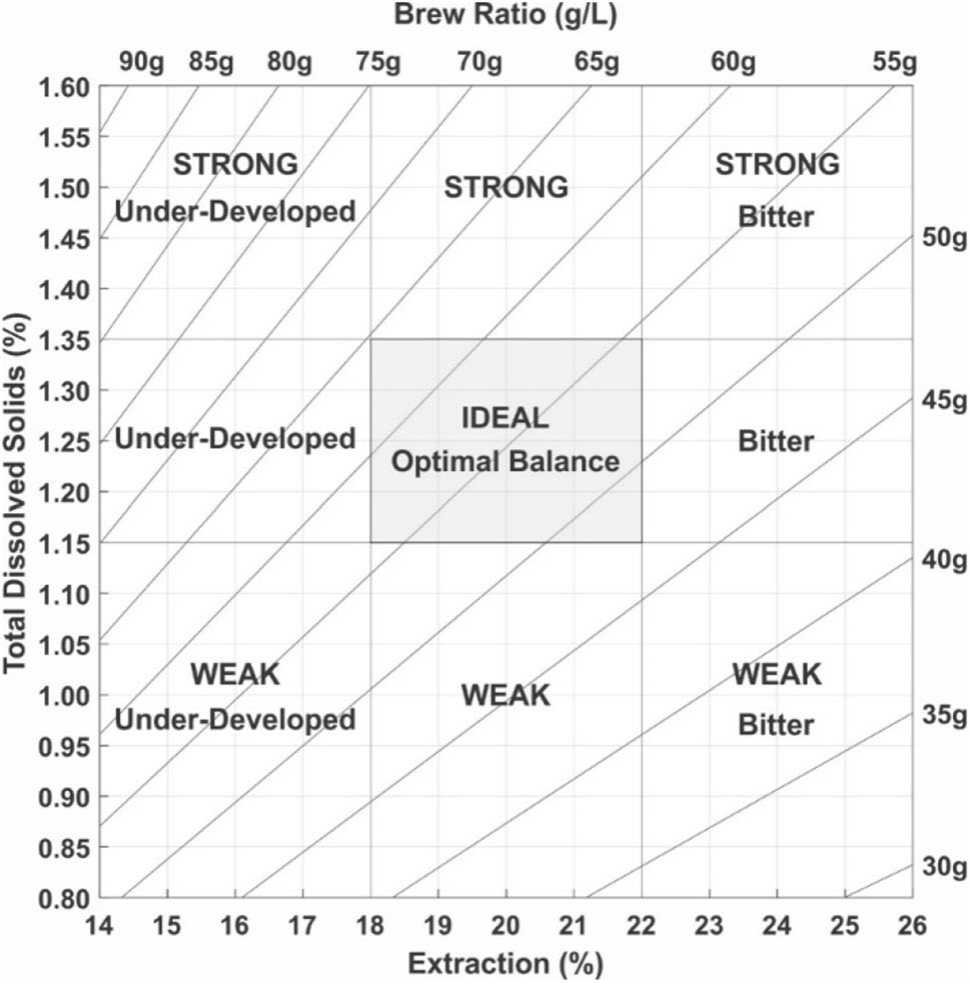
The classic Coffee Brewing Control Chart, originally developed by Lockhart^2^. Reproduced from Lingle^4^.

Coffee’s widespread consumer popularity is driven heavily by sensory quality^1^. Accordingly, early researchers established key metrics to characterize the quality of the brew. Seminal work by Lockhart in 1957^2^ related the quality of brewed coffee to two key metrics: the “brew strength,” which is the mass fraction of soluble solids in the brew (commonly characterized as the total dissolved solids, or TDS), and the “extraction yield,” which is the mass fraction of soluble solids removed from the coffee grounds (commonly referred to as the percent extraction, or PE). These quantities, which are linked via conservation of mass to the brew ratio of water to coffee grounds^3^, were combined by Lockhart in the classic “Coffee Brewing Control Chart” (Fig. 1). This chart still serves as the basis of vocational training in the coffee industry, as exemplified by the Coffee Brewing Handbook published by the Specialty Coffee Association^4^. The chart is divided into 9 regions, with vertical demarcations versus TDS labelled as “strong” or “weak” and horizontal demarcations versus PE labelled as “bitter” or “underdeveloped”. The chart’s central region, which spans TDS values of 1.15–1.35% and PE values of 18–22%, is denoted as “ideal”, and is identified as the Specialty Coffee Association’s “Golden Cup Standard”^5^.

Although widely used, the Coffee Brewing Control Chart is problematic from a modern sensory methodology perspective. It conflates hedonic or quality descriptors (“ideal”), measures of scale (“weak”, “strong”), and descriptive language (“bitter”). It is also unclear what consumer populations find coffees in the centre of the chart to be “ideal.” Perhaps most importantly, the chart omits the wide variety of sensory attributes possible in coffee. World Coffee Research’s Sensory Lexicon^6^ and the new Coffee Taster’s Flavor Wheel^7^ list more than 100 different flavour and aroma attributes that have been detected in black coffee, including many attributes considered desirable (e.g., caramel, berry, dark chocolate, hazelnut) or undesirable (e.g., rubber, medicinal, mouldy).

In the years since the original publication of the brewing control chart, our understanding of the factors that impact this tremendous variety of possible flavours in coffee has grown. Green and roasted coffee have been chemically characterized and we know that coffee taste and flavour are made up of a complex mixture of carbohydrates, acids, lipids, proteins, antioxidants, and volatile aroma compounds^8^, which can be manipulated at all steps, from origin, including the terroir of the coffee cherry, to the post-harvest processing method, the roasting, and the final brewing methods^9^. Focusing on the latter, espresso, lungo, filter (drip) coffee, and French press, among others were found to have differing flavour intensities, with espresso type extractions being higher in roasty, fruity, bitter, and astringent attributes, whereas filter coffee and French press were found to be sweeter; TDS and PE were not controlled, however, and espresso type coffees are much stronger, with a higher TDS^10^. Various studies have looked at the impact of brewing extraction time on sensory quality, with Lee et al. demonstrating that 50% of measured coffee components extract within the first quarter of brewing volume but that some flavour-active compounds extract more slowly, notably those like methyl cinnamate and indole that are less soluble in water than fast extractors like caffeine^11^. Mestdagh et al. investigated the kinetics of aroma extraction, showing a correlation between polarity of aroma compounds and extraction speed^12^.

The drip brew basket geometry plays a role in the sensory properties of the coffee, with a flat-bottomed drip brew basket yielding a lower TDS and many different sensory properties than a semi-conical drip brew basket^13^. Batali et al. examined the role of TDS via temporal fractionation of a drip brew, and found that attributes like bitter, sour, and smoky were positively correlated with TDS, while attributes like sweet, fruity, and floral were negatively correlated with TDS^14^. Most recently, Frost et al. characterized the impact of TDS and PE on sensory quality of drip brew for three different roast levels, and found similar trends—generally more bitter and sour at higher TDS, and sweeter at lower TDS, regardless of roast level^15^.

Notably, none of the above work examined the effect of the brew temperature on the sensory quality of coffee, although it is widely argued that brew temperature plays a key role. The Coffee Brewing Handbook published by the Specialty Coffee Association states that a water extraction temperature of 92–96 °C is necessary for “proper extraction” (i.e., a TDS and PE within the “ideal range”) to occur^4^. This standard has heavily influenced the home brewer market: for certification, the Specialty Coffee Association requires that brewers “…reach 92 °C within the first minute [of the brew], maintain at least that temperature for the remainder of the brew cycle, and never exceed 96 °C”^16^. A variety of published coffee books describe similar temperature ranges as optimal^8,17–19^. None of these sources, however, provide any data in support of the specified temperature ranges.

In the scientific literature, it is accepted that temperature impacts the solubility, volatility, and extraction kinetics of various coffee components^12,20^, but there is only a small amount of existing scientific literature on the direct impact of brewing temperature on coffee sensory quality. At one extreme, cold brew coffee prepared at temperatures less than 25 °C is widely reported to be sweeter and less acidic than hot brewed coffee^20–23^, suggesting that less dramatic temperature differences might play a similar role for hot drip brew coffee. Early work by Pangborn examined coffee brewed using a stirred pour-over technique with distilled water at 65, 80, 90, and 100 °C, and found that bitterness and sourness in general increased with temperature; complementary experiments with hard and soft water at 80 and 100 °C yielded similar increases^24^. At hotter temperatures, closer to the traditional range of drip brew, an investigation of espresso coffee systematically varied the water temperature between 88 and 98 °C and found that hotter water predictably increased TDS and PE, and yielded more acrid, roasty, bitter, and sour attributes^25^. Further work on espresso determined that espressos prepared with sufficiently small temperature differences (80–93 °C) yielded imperceptible differences as assessed by triangle test performed by untrained panellists, while larger temperature differences (80–128 °C) yielded a significant number of panellists able to discern difference^26^. Some differences were also observed when espresso was prepared using a temperature gradient (ramping up or ramping down between 88 °C and 93 °C) compared to a fixed temperature (90 °C), with the “ramp up” gradient espressos evaluated more favourably by panellists than the espressos prepared with fixed or “ramp down” gradients in brewing temperature^27^. No published work to date has examined the effect of brew temperature on the sensory qualities of drip brew coffee.

An important potential confounding factor in any analysis of the effect of brew temperature is that it complicates control of the serving temperature, which is well known to impact sensory perception^28^. Coffees served at higher temperature were reportedly perceived as more roasty and bitter than those served at lower temperatures, while most other attributes decreased in perceived intensity at higher serving temperatures^29,30^. Lower temperature coffee samples are also generally rated less favourably by untrained consumers^31,32^. Ultimately, temperature is believed to be highly relevant to coffee in terms of extraction, but to date no direct comparison of the effect of brew temperature at equivalent TDS and PE has been made and evaluated at controlled serving temperature.

Aside from sensory quality, another key consideration regarding brew temperature is energy consumption. The brewing step has been shown to have the highest carbon footprint of the coffee supply chain, accounting for considerably more energy usage than all other steps of the supply chain, including farming and roasting^33^. Therefore, understanding the impact of brew temperature could potentially reduce the energy consumption of the coffee industry if it is determined that the brewer standards are held to an unnecessarily high temperature.

In this study, we investigated the sensory differences in hot-brewed drip coffee prepared at different brew temperatures but at equivalent TDS, PE, and serving temperatures. Our primary goal was to advance understanding as to how these brewing variables impact the sensory quality of coffee. In particular, we sought to determine how specific sensory attributes, such as those listed in the Coffee Taster’s Flavor Wheel, are affected by extraction under different conditions. Towards these aims, we brewed the same roasted coffee at nine distinct positions across the Coffee Brewing Control Chart, at three different water temperatures (87 °C, 90 °C, and 93 °C), for a total of 27 sample types. The grind size and flow rate were adjusted as needed to yield brews at the same TDS and PE when prepared with different water temperature, such that the TDS, PE, and brew temperature were all independent factors. The coffee samples were evaluated blind and in triplicate by descriptive analysis with a group of trained sensory panellists to determine the intensities of 31 different sensory attributes (listed in Table 1). The trends observed in attribute intensity offer a more substantial understanding of the effect of extraction and brewing parameters, including brew temperature, on the sensory quality of drip brew coffee.

**Table 1:** Sensory attributes and corresponding references for panellist training.

Type	Attribute name	Reference standard
Aroma	Floral	Dried *Celestial Seasonings* chamomile tea
	Green/vegetative	Cut fresh cucumber and snap peas, green beans
	Nutty	Hazelnut oil
Taste	Bitter	**0.1% caffeine solution**
	Salty	**0.15% iodized salt solution**
	Sour	**1.25% citric acid solution**
	Sweet	**2% sucrose solution**
Flavour by mouth	Ashy	Spent *Camel* cigarette ash
	Berry	Mixed frozen berries defrosted
	Black pepper	Ground black pepper
	Black tea	*Lipton* brewed black tea
	Brothy	1 tsp *Better than Bouillion* vegetable stock in hot water
	Brown roast	*Starbucks* French roast coffee
	Brown spice	Equal parts dried ground cinnamon, nutmeg, cloves
	Brown sugar	*Signature Select* light brown sugar
	Cereal	Unsweetened *Chex* breakfast cereal
	Citrus	Sliced lemon and grapefruit
	Dark chocolate	*Hershey’s* Cocoa Powder, 90% *Ghirardelli* dark chocolate
	Dark green	*Green Giant* cut green beans
	Dried fruit	*Sunsweet* dried prune and prune juice
	Earthy	Wet potting soil
	Fermented	*GT’s Enlightened* Organic Raw Unflavored Kombucha
	Fresh green	Cut fresh cucumber and snap peas
	Fruity	*Tree Top* Applesauce
	Molasses	*Grandma’s Original* Molasses
	Nutty	Equal parts raw hazelnut, almond, walnut, and cashew, well mixed
	Papery/musty	Damp cardboard
	Rubber	Rubber band, pencil eraser
	Smoky	*Wright’s* liquid smoke
	Tobacco	*Camel* cigarette tobacco
	Woody	Wood chips, cedar ball
Mouthfeel	Astringent	**2% alum solution**
	Viscous (body)	**5% starch solution**

Bolded references were aqueous solutions (mass percent) consumed by mouth; all others were aroma references.

## Results

### Brewing and extraction

**Figure 2:**
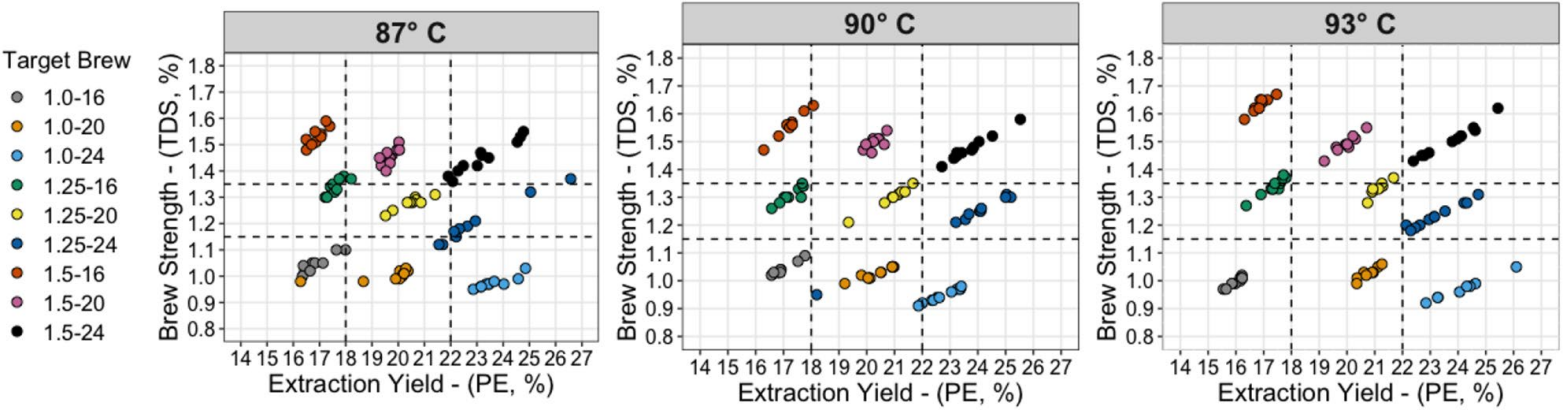
Coffee brewing extraction profiles measured during sensory trials (n = 9 for each target brew, N = 270 total brews), at three different brew temperatures. Code in legend denotes TDS-PE target value. Vertical and horizontal dashed lines denote the zones in the classic Coffee Brewing Control Chart (cf. Fig. 1). See Supplementary Table S1 for all brewing parameters and statistical measures.

Brew time, flow rate, dose (i.e., amount of ground coffee), and grind size were systematically varied to control extraction with the same coffee, using the same type of commercial drip brewer, at different brew temperatures. Figure 2 shows the measured TDS and PE for each brewed coffee sample assessed in this study (a total of 270 separate brews). Although there was some amount of variation from brew to brew at each target condition, presumably due to channelling, the standard deviations of the TDS and PE at each target condition were small compared to the differences among the target values. The brew-to-brew variability was slightly larger in higher PE brews, which generally required a longer brew time and may accordingly have had less consistency from varying amounts of channeling. Because of this variation in brew precision, two statistical analyses were performed: (M)anova treated TDS and PE as discrete factor levels, while Response Surface Methodology used measured TDS and PE instead of target TDS and PE, to ensure that conclusions were consistent using both methods. All brewing parameters and resulting means and standard deviations of the TDS/PE and standard deviations are listed in Supplementary Table S1. All coffees were served at measured temperatures of 65 ± 1 °C.

### Multivariate and univariate analyses of variance

Overall, the sensory descriptive analysis results exhibited a pronounced dependence on TDS and PE, but little dependence on brewing temperature. Multivariate analysis of variance showed significant differences (α = 0.05) for our dependent variables (the sensory attributes) based on TDS, PE, and TDS x PE interaction (Table 2). At the multivariate level, there was no significance shown based on temperature or temperature interactions at any level less than 4-way interactions. The full MANOVA results, including 4-way interactions, are shown in Supplementary Table S2.

**Table 2:** Table of select F-ratios and p values from multivariate analysis of variance of independent factors affecting sensory attributes, bolded with asterisks to indicate statistical significance with p < 0.05.

Factor	F-ratio	p-value
Temperature	1.25	0.11
TDS	**5.44**	< 0.001*
PE	**2.46**	< 0.001*
Temperature: TDS	1.16	0.14
Temperature: PE	1.07	0.30
TDS: PE	**1.56**	< 0.001*
Temperature: TDS: PE	1.10	0.17

For complete MANOVA details, see Supplementary Table S2; for univariate 5 way ANOVA, see Supplementary Table S3; and for pseudomixed ANOVA, see Supplementary Table S4.

**Figure 3:**
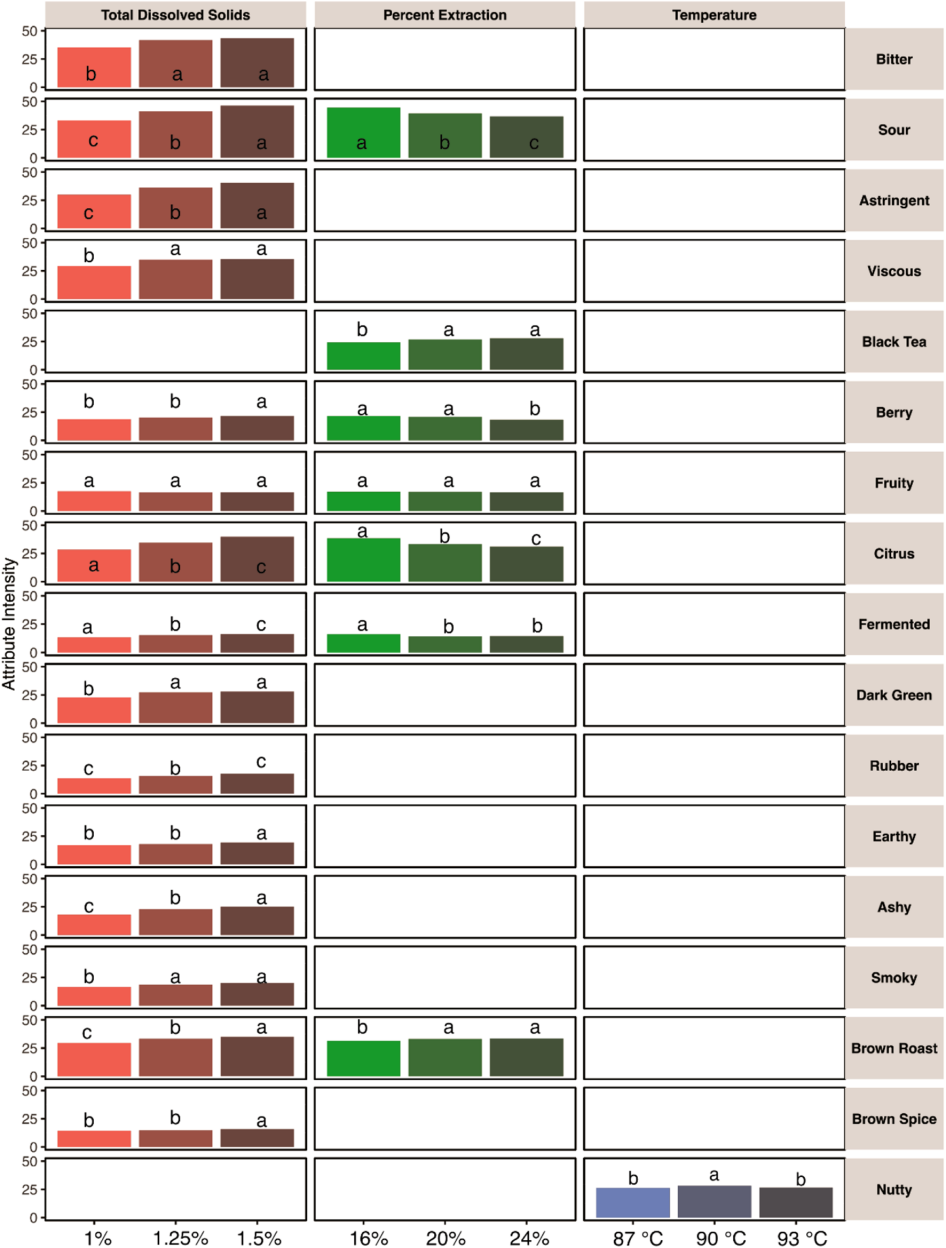
Column plots of the mean score for each sensory attribute found significant in ANOVA by independent variable, with Fisher’s LSD letter codes indicating variable groupings. Blank entries denote no statistically significant variation. Attributes arranged in order consistent with the Coffee Taster’s Flavor Wheel^7^. See Supplementary Table S5 for all numeric mean values and LSD values represented here.

Examination of the corresponding univariate statistics using the 5-way ANOVA with 4-way interactions showed that 17 of 31 tested attributes varied significantly based on TDS, PE, brewing temperature, and/or TDS × PE interaction. Figure 3 shows column plots of all attributes found significant with intensities averaged for each variable across the two other independent variables (i.e. average intensities for all PE and temperature levels at 1% TDS, etc.), with letter codes to show significance; blank entries in the figure denote no significant difference. Although no significance was observed at the multivariate level for brew temperature, one of 31 tested attributes (*Nutty*) had significant differences based on brew temperature at the univariate level. In contrast, eight attributes (*Bitter, Astringent, Viscous, Dark Green, Rubber, Earthy, Ashy, Smoky,* and *Brown Spice*) had significant differences based on TDS alone, one attribute (*Black Tea*) had significant differences based on PE alone, and five attributes (*Sour, Berry, Citrus, Fermented,* and *Brown Roast*) had signficant differences based on both TDS and PE.

**Figure 4:**
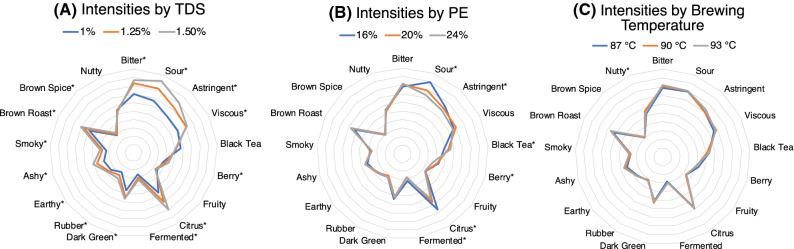
Spider web plots visualizing differences in attribute intensities by all variables significant for at least one of the three factors, for (**A**) TDS, (**B**) PE, and (**C**) brew temperature. Asterisks indicate statistical significance (p < 0.05). Inner ring corresponds to attribute intensity of 0, while the outer ring corresponds to an attribute intensity of 50; intermediate rings are spaced evenly in increments of 5 intensity points.

These trends can also be visualized using spider web plots to indicate mean differences for overall TDS, overall PE, and overall temperature for the 17 specific attributes that varied significantly for at least one factor (Fig. 4). Note that the shape of the spider web plot shifts considerably for different TDS values (Fig. 4a), with the most pronounced changes for *Bitter* taste, *Sour* taste, and *Astringent* mouthfeel, and smaller but statistically significant shifts for 13 other attributes. The shape of the spider web plot shifts to a lesser extent for different PE values (Fig. 4b), with the largest differences observed for *Sour*, *Black Tea*, and *Citrus*. Finally, there are almost no perceptible differences in the shape of the spider web plot for different brew temperatures (Fig. 4c), with only *Nutty* flavour yielding a slight but statistically significant shift with temperature.

Several significant interactions were also seen in the univariate ANOVAs. *Sour*, *Berry*, and *Fruity* showed a significant TDS × PE interaction. *Brown Spice* showed a Temperature × TDS interaction, and *Earthy* and *Fermented* showed a Temperature × TDS × PE three-way interaction. These interactions were all detected in attributes that exhibited relatively low magnitudes of change. Notably, these temperature interactions are only significant in the univariate ANOVA, not in the multivariate ANOVA. All interactions are shown in Supplementary Fig. S1.

### Principal component analysis

**Figure 5:**
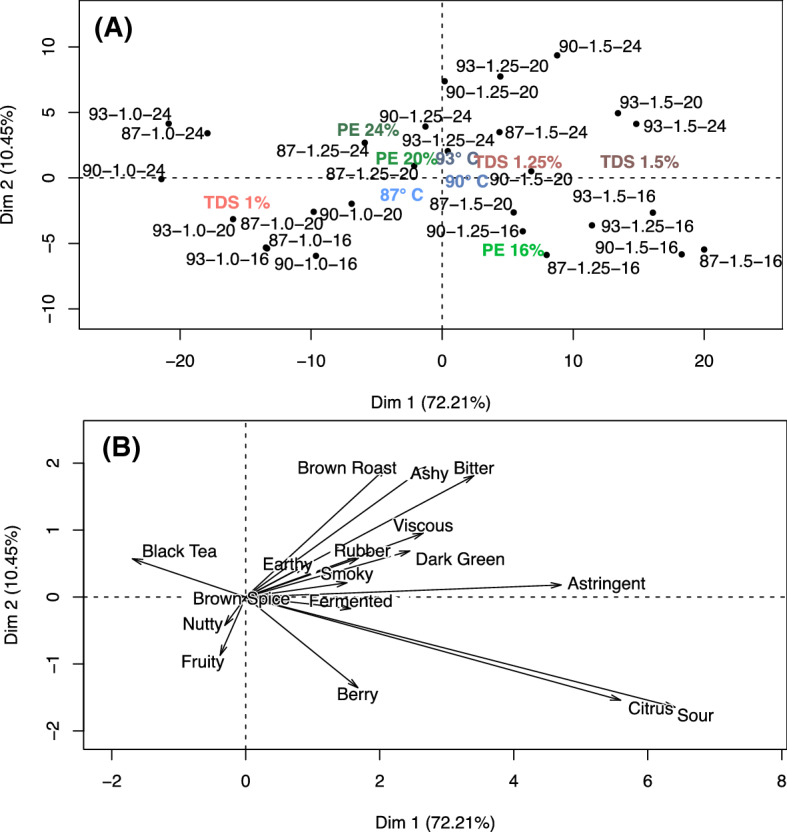
(**A**) Principal Component Analysis of individual brews, with each numerical code indicating the corresponding “temp–TDS–PE” combination of that brew. The independent variable means are also plotted and colour coded as follows: TDS, red; PE, green; and brewing temperature, blue; all are scaled from light to dark in accordance with variable magnitude. (**B**) Corresponding map of the attribute loadings for the dependent variables (sensory attributes).

The spider web plots in Fig. 4 consider the overall means of the sensory attribute intensities. To examine the relationship among the attributes, we performed principal component analysis (PCA) for all significant attributes. Figure 5a shows clear separation of individual samples across the product space. Dimension 1 encompasses 72.2% of the variance, and dimension 2 encompasses 10.5%. The TDS spreads primarily across dimension 1, with low (1%) TDS samples generally on the left side of the product space, moving to intermediate (1.25%) and high (1.5%) TDS samples towards the right side of the product space. The PE spreads primarily across dimension 2, with low (16%) PE samples primarily in the bottom half of the product space, and high (20 to 24%) PE samples in the top half of the product space. The difference in percent explained between dimension 1 and dimension 2 indicates that TDS is a much more significant driver of variance in the product set than PE. No clear trends with respect to brew temperature are observed across the product space. The brew temperature variables are clustered tightly around the middle of the product space, indicating that temperature is not a major driver of sensory differences.

Figure 5b shows the dependent variables (i.e., the sensory attributes) and demonstrates the drivers of difference among all the samples in Fig. 5a. The intensities of *Black Tea*, *Nutty*, and *Fruity* increase in the direction of lower TDS (cf. left-hand side of Fig. 5a), whereas all other significant attributes tend to increase in the direction of higher TDS. Likewise, the intensities of *Sour, Citrus, Berry,* and *Fruity* all increase considerably in directions of lower PE, whereas almost all other attributes—including most notably *Bitter, Ashy*, and *Brown Roast,* increase in directions of higher PE.

### Response surface methodology

**Figure 6:**
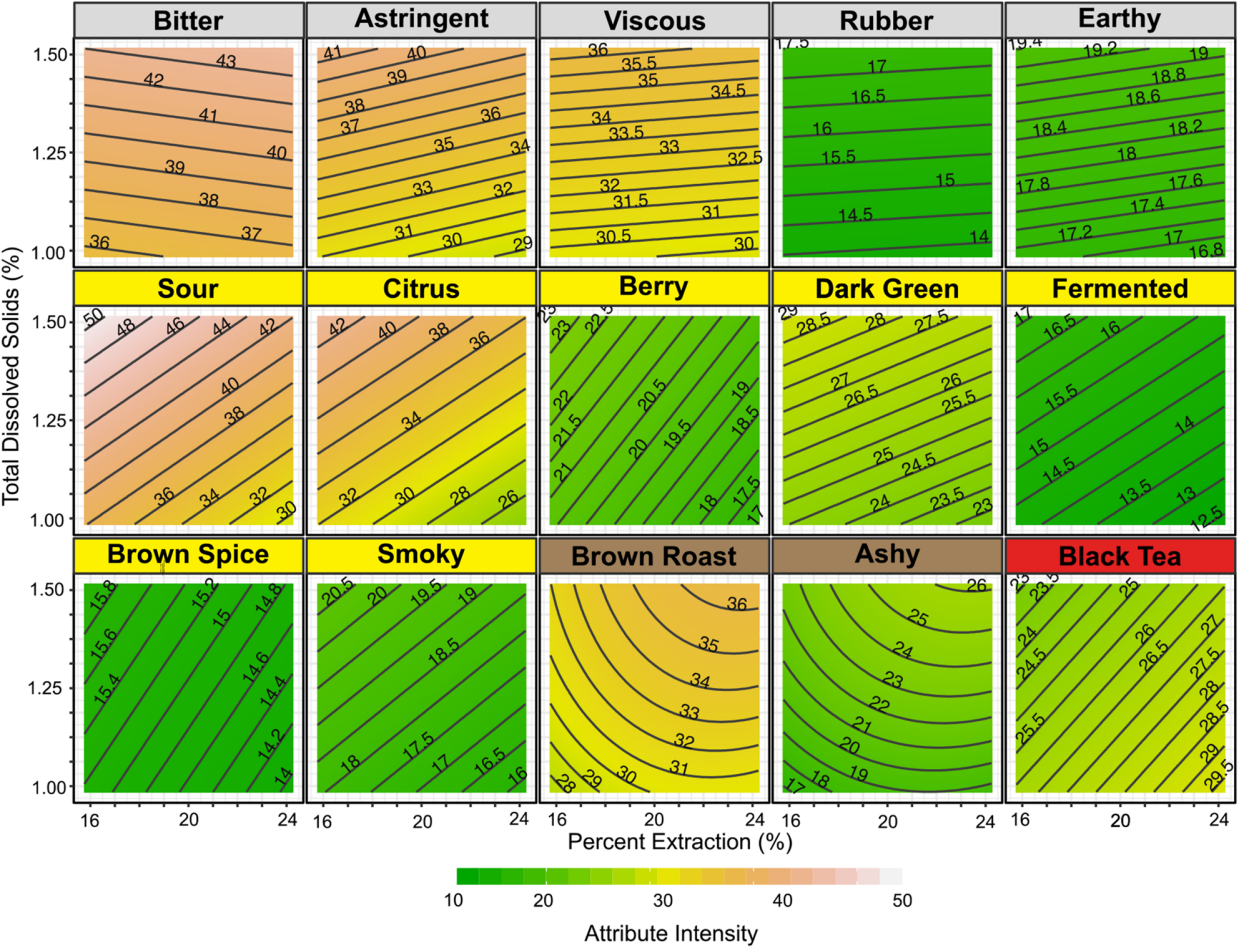
Contour plots of fitted response surface for attribute intensity versus total dissolved solids and percent extraction for all sensory attributes found to vary significantly by ANOVA. Grey attribute titles indicate positive correlation with TDS only; yellow titles indicate a positive correlation with TDS and negative correlation with PE; brown titles indicate a second order fit increasing with both TDS and PE; and red title indicates a negative correlation with TDS and positive correlation with PE.

The PCA plots show indirectly the relationship between the independent variables and the driving sensory attributes. To plot these relationships more directly, we generated three-dimensional models of the attribute intensities versus TDS and PE using Response Surface Methodology (RSM). The resulting RSM contour plots (Fig. 6) mimic the presentation of the classic Coffee Brewing Control Chart (cf. Figure 1), but each focuses on a specific sensory attribute. Of the attributes significant in the ANOVA, 15 returned a significant first order (linear) or second order (quadratic) fit when Response Surface Methodology was applied. Because of the lack of significance of temperature in the MANOVA, the data from all brewing temperatures were modelled together. Several attributes (*Earthy, Fermented,* and *Brown Spice* flavours) did have a significant temperature interaction by ANOVA, so the four-dimensional models are shown in Supplementary Fig. S2.

The 15 attributes can be characterized as exhibiting four different types of behaviour (Fig. 6, colour coded). The attributes *Bitter, Astringent, Viscous, Rubber,* and *Earthy* all increased linearly with TDS, with no statistically significant correlation with PE. The slight observed slopes with respect to PE in these plots are not statistically significant correlations. In contrast, the second type of behaviour included *Sour*, *Citrus*, *Berry*, *Dark Green, Fermented*, *Brown Spice,* and *Smoky*, all of which increased strongly with TDS but had a negative correlation with PE, with the maximal intensities located at top left (high TDS, low PE). Third, the attributes *Brown Roast* and *Ashy* were the only attributes that yielded a statistically significant second order fit, with both maximal at high TDS, high PE, but the curved fit indicates that for both attributes PE makes little difference beyond a certain threshold. Finally, one attribute only, *Black Tea*, decreased with TDS (i.e., it was negatively correlated to TDS) and increased with PE, such that its maximum was at the bottom right (low TDS, high PE).

## Discussion

### Impact of brewing temperature on sensory profile

The most surprising aspect of the results presented here is the insensitivity to the brew temperature. MANOVA tends to be more robust to Type I error than univariate ANOVAs^34^ and indeed the MANOVA analysis (see Table 2) indicates no significant effects with respect to temperature up to three-way interactions. The univariate ANOVA shows only slightly different results. Figure 3 shows that of 31 tested sensory attributes, we observed only a very slight increase in one attribute, *Nutty* flavour perception, at the 90 °C water temperature compared to the higher and lower temperature brews. This difference in means was only one point on a scale out of 100, however, which is very small compared to the sizable differences in means noted for other attributes. Although the difference in *Nutty* flavour was found to be statistically significant (at p < 0.05), further testing would be necessary to confirm that this result was not simply a Type I error. Furthermore, ANOVA univariate temperature interactions were significant statistically but small in magnitude. *Brown Spice* flavour showed a temperature × TDS interaction, *Fermented* and *Earthy* flavours both had a three-way TDS × PE × temperature interaction, as visualized in Supplementary Fig. S1. The magnitude of the difference in each case was small (each less than 5 points on a 100 point scale). We conclude that within the range measured, temperature had a negligible impact on the perceptible sensory quality of the brewed coffee when TDS and PE were kept constant.

Our data was collected with a controlled consumption temperature, but realistically service temperature would not be precisely measured nor controlled in many service industry scenarios. Therefore, it is worth noting that temperature could play a role in sensory quality if coffees brewed at different temperatures are then served and consumed at different temperatures. A recent study has shown bitter, chocolate, roast, and ashy to increase with consumption temperature, but sour taste decreases with increasing consumption temperature^30^. When investigating multiple coffee types, Adhikari, Chambers, and Koppel showed that while attribute intensities increased with increasing consumption temperature, the flavours impacted by temperature depended on the coffee type^35^. This potential confounding effect is an unavoidable difficulty in sensory studies on a hot beverage. Also, logistical challenges in brewing and serving a large amount of coffee at the correct serving temperature meant that the coffee was served approximately 30 min post brew, which may have allowed for a change in flavour that impacts the sensory profile in comparison to what might be observed if served and cooled immediately. While it is known that chemical composition of brewed coffee changes with time^8^, and coffee held in an open carafe on a hot plate will change in sensory quality^36^, we are unaware of published data regarding changes in the sensory quality of brewed coffee held in vacuum insulated carafes over the scale of 30 min. Future work is necessary to investigate this hypothesis.

### Impact of total dissolved solids and percent extraction on sensory profile

In contrast to the lack of impact of brew temperature, our results confirm that both the TDS and PE have significant impacts on the sensory qualities of the brew. Although the idea that TDS and PE affect the sensory qualities of brewed coffee was originally formulated in the 1950s, our work provides new insight as to how specific and important sensory attributes vary with brewing conditions. The TDS had the greatest impact on the sensory properties, with PE yielding a lesser but noticeable impact, as visualized in Figs. 3 and 4 especially. An important point is that higher PE in general is achieved by longer residence time (cf. brewing parameters in Supplementary Table S1), compared to the low PE samples, which involve much shorter brew times. Previous published work has indicated that many, and potentially the majority, of components in a coffee drip brew are extracted early and then plateau in terms of total amount removed from the coffee grounds, i.e., their individual extraction percentages plateau early in the brew^11,12,14,37^. Thus, continued addition of water after all of these ‘fast extractors’ are removed from the solid grounds will serve to dilute their concentrations in the final brew, while providing more opportunity for the ‘slow extractors’ to continue extracting from the coffee grounds. The sensory profile will ultimately be governed by the relative ratios of the respective concentrations of the fast and slow extractors in the final brew.

More specifically, the RSMs in Fig. 6 help visualize different trends exhibited by different sensory attributes. Most attributes had a first order planar fit, aside from ashy flavour and brown roast flavour. Five attributes increased with TDS only—*Bitter, Astringent, Rubber, Earthy,* and *Viscous*. Some of these attributes appear to have a small relationship with PE, but when considering Fig. 3 and the ANOVAs, we emphasize that the relationship with PE for these specific attributes was not statistically significant. In particular, the results regarding *Bitter* taste differ with the classic brewing control chart, which implies that bitterness is maximized at high PE values (cf. Fig. 1)^2^. A more recent study suggests that *Bitter* taste and *Viscous* mouthfeel increase with both TDS and PE when generalized across multiple different roast levels, though the dependence changed with individual roasts^15^.

Although we did not perform any chemical analysis here, previously reported measurements provide insight on possible mechanistic interpretations of the observed sensory profiles. *Bitter* and *Astringent* are attributes potentially related to chlorogenic acids, as work has shown a correlation between these compounds and perceived bitterness and astringency^10^, but further chemical analysis would be required to confirm this relationship in our work as well. *Viscous* is an attribute that would be expected to scale linearly with TDS, as it refers only to the thickness and mouthfeel of the coffee, and a coffee with more dissolved solids should accordingly have a thicker mouthfeel. *Rubber* and *Earthy* flavours are possibly related to sulphur containing compounds, such as thiopenes and thiazoles^38^. While our results suggest that differences in *Rubber* and *Earthy* were small (less than 5 points on a scale of 0–100, as shown by RSMs in Fig. 6), it should be noted that the coffee used here was *Coffea arabica*, which is known to have a lower concentration of thiopenes and thiazoles compared to *C. canephora*^39^*.* Although further research is necessary to confirm that our results are generalizable to different coffee types, we speculate that similar trends will be more pronounced when brewing with *C. canephora* in which sensory attributes such as rubber or earthy are likely more substantially present in the bean.

The next category of attributes involved those that increased with TDS but decreased with PE: *Sour, Citrus, Dark Green, Smoky, Brown Spice,* and *Fermented*. *Sour* taste is a particularly important attribute: of all the attributes measured, it exhibited the largest change throughout the measured variables, with a 20-point difference in mean intensity between high TDS, low PE and low TDS, high PE (Fig. 6). These patterns are possibly due to the relatively fast extraction of organic acids in coffee, which is high in acids such as acetic acid, quinic acid, malic acid, and others that are highly soluble and will rapidly extract into brewed coffee^40,41^. Several of these other attributes that follow the same pattern might be acid-associated flavours such as *Citrus* (citric acid). *Fermented* could arguably reflect panellist perception of acetic acid, normally associated with vinegar (also a product of fermentation), and one found in high concentrations in kombucha, the chosen sensory standard for this study^42^. We emphasize that the fermented flavour present in the coffee might or might not be due to the presence of naturally occurring acetic acid in the coffee, and further chemical characterization would be necessary to corroborate this hypothesis. *Berry* might also be a flavour that is associated with sourness, considering the sensory standard used in this study (unsweetened fresh mixed berries) is a product typically considered tart/sour and high in malic and citric acids^43^. These cross modal interactions between the taste and aroma components of flavour would serve to enhance both the *Sour* taste and the *Fermented*, *Berry*, and *Citrus* flavours, consistent with the observed pattern^44^. The decrease with PE is likely due to other attributes that continue to increase in intensity due to slower extraction times, such as bitter compounds that have the same concentration at a particular TDS regardless of percent extraction. In other words, the PE likely alters the relative ratio of bitter compounds to acids, and thus the perceived taste.

Two sensory attributes, *Brown Roast* and *Ashy* flavours, both followed second-order fits that generally increased towards high TDS and high PE. The second order fit indicates that PE is only significant for these attributes below a certain threshold PE value, above which the attribute intensity remains unchanged at fixed TDS. Though the chemical rationale for this behaviour is obscure without further study, the extraction pattern of these flavours might be a contributor to the decrease in perception of sourness at a higher percent extraction, with these flavours at high PE masking the acidity of the high TDS coffees. There is little data related to this sort of phenomenon with these kinds of flavours and tastes; odour-taste interactions have been investigated for other attributes in coffee^45^, and sourness in other studies has been shown to be easily suppressed^46^, but there is much room for future studies to continue to investigate the way flavours change and interact in coffee.

One attribute, *Black Tea*, followed a unique pattern, peaking in intensity at low TDS, high PE. This behaviour was possibly due to black tea flavour being related to aromatic floral volatile compounds, which could potentially be linked to β-damascenone, a compound known for its floral aroma. This molecule has been shown in kinetic studies to extract more slowly^12^, so it possibly increases in relative proportion to other flavour compounds during the longer brews associated with higher PE. Because some popular and valuable specialty coffee types, such as the Panamanian Geisha coffee, are associated with “flowery”^47^ and tea-like^48^ flavours it is in the coffee industry’s interest to understand how to brew to highlight those attributes.

We emphasize that all of these potential connections between the chemical composition of a coffee brew and the resulting sensory properties are informed hypotheses, and more detailed chemical analysis of flavour extraction across the Coffee Brewing Control Chart is necessary. Furthermore, there are many different methods of brewing coffee, but this research only investigated drip brewing using a commercial brewer chosen for its versatile programmability. The resulting data could nevertheless be useful to inform the design of automatic brewers as well as the choices of hand brewers to control desired flavour profiles, using the results and procedures in Supplementary Table S1. We hypothesize that similar relationships between TDS and PE and sensory quality would be observed in all types of drip brew, but we emphasize that the precise procedures necessary to obtain a target TDS and PE would vary by brewer and coffee type. We treated TDS and PE as independent variables for the purpose of this experimental study, but in practice TDS and PE themselves depend on many factors in a complicated fashion (cf. Supplementary Table S1). A more fundamental understanding of how truly independent variables like flow rate, grind size, and brew ratio impact TDS and PE would provide better insight on how to best modulate the sensory profile of the coffee.

### Implications for the coffee industry

Our results have several important implications for the coffee industry. First, the classic Coffee Brewing Control Chart (cf. Fig. 1) is widely featured in brewer education, but our results suggest that its present configuration could be misleading. For example, the chart implies that “Bitter” increases with PE, whereas bitterness and several other attributes were found here to increase solely with TDS. Likewise, “Strong” and “Weak” as terms on the chart could be misleading, since our data suggest there is no position on the chart where all attributes are strongest or all attributes are weakest, and indeed a highly prized attribute—black tea—was strongest at low TDS. The term “Underdeveloped,” likely reflects the increased sourness and associated flavours observed here, suggesting the language should be reconsidered to emphasize specific and more relatable sensory attributes.

The most prominent feature within the classic Coffee Brewing Control Chart is arguably the “Ideal” box. We emphasize that our study was purely quantitative (i.e., sensory descriptive analysis rather than hedonic preference), but previous studies have identified attributes that are drivers of liking or disliking for consumers. Research from Frost et al. has indicated that a majority of consumers dislike bitterness and astringency in coffee products, whereas attributes like floral are positive drivers of liking^13^, which might indicate that the bottom third of the coffee brewing control chart (“Weak”, cf. Fig. 1) might be more “ideal” for many consumers. However, the same study has indicated that “roasted” flavour is also a driver of liking, and other work has identified significant segments of coffee consumer populations are drawn towards bitter and astringent coffee^49^. There is relatively little data on coffee consumer preference for specific flavours in coffee, but these observations suggest that the “ideal” box within the classic control chart is not necessarily ideal for everyone and accordingly might not be a useful descriptor for coffee brewing.

Our results also suggest that the current industry guidelines focused on brew temperature might be misplaced. For example, home brewers currently fail certification if they fail to maintain their temperature at 92–96 °C^16^, but our data indicate that coffees brewed at temperatures as low as 87 °C were indistinguishable from those brewed at 93 °C (provided the grind size, brew ratio, and flow rate were adjusted to hold the TDS and PE constant). This observation suggests that brewer certification requirements might focus more on precise control of flow rate over a wider range of temperatures.

One potential benefit from these results is that lower brewing temperature offers increased consumer safety. As Supplementary Table S1 shows, coffee temperature immediately post brew was 4–5 °C lower for the 87° brewing temperature than for the 93° brewing temperature. This lower temperature is safer with regard to the risk of scald burns^50^. Furthermore, although coffee as a beverage has been removed from lists of substances believed to be carcinogenic due to chemical composition^51^, studies of tea and maté consumption indicate that regular consumption of beverages above 65 °C is correlated to increased risk of oesophageal cancer^52^. Brewing coffee at lower temperatures might help protect against this possible risk.

Additionally, lower brew temperature also has an important consequence in terms of environmental sustainability. Since a cup of coffee is approximately 99% water, the energy required to heat the water represents a large fraction of the overall energy usage in the entire coffee supply chain. At least one estimate indicates that energy usage in a café accounts for 45% of the overall carbon dioxide emissions of brewed coffee production, even when accounting for all other steps including the agronomy at the farm, the post-harvest processing, and the roasting^33^. Decreasing the average brew temperature could thus have an outsize impact on overall sustainability efforts in coffee. The results presented here suggest that such improvements to consumer health and the global environment could be obtained without sacrificing the sensory quality of the brewed coffee.

Finally, further investigation is needed to characterize how TDS and PE impact sensory quality in different brewing methods, such as full immersion and espresso. Future work should compare full immersion cold brew and hot brew to ascertain whether higher temperature differences yield larger differences in sensory quality. Likewise, espresso reaches a much higher TDS than other brew methods and likely is much more sensitive to temperature and pressure during the very rapid extraction. The results presented here for drip brew will provide insight for sensory investigations in these other brewing techniques.

## Conclusion

Overall, our study yielded two key results. First, we found that when extraction is controlled through other means, and over the range we tested, the temperature of the brewing water plays a minimal role in the sensory properties of the coffee as measured by a trained descriptive analysis panel. Second, we report how specific sensory attributes vary with brewing parameters, with brew total dissolved solids effecting more perceptible differences than percent extraction. We anticipate that these results will help coffee professionals better extract the flavours that they seek, and also help inform sustainability efforts aimed at energy minimization during brewing.

## Materials and methods

### Coffee

Wet-washed *Coffea arabica* varietals comprising a mixture of Bourbon, Catuai, Caturra, Lempira, Ihcafe 90, Pacas, and Typica varietals from Marcala, La Paz, Honduras were provided by Royal Coffee in Oakland, CA. Green coffee was mixed then roasted in a Loring S35 Kestral in 29 kg batches for an 11:29 (minutes:seconds) total roast time, with first crack recorded at 9:00 and a 2:30 development time (see Supplementary Fig. S3 for roast profile). All roasting was performed in the same day, and batches were combined and well mixed post-roasting to control for any variation among roasts. One week after roasting to allow off-gassing, coffees were packaged in 1 kg bags, vacuum sealed, and stored in a − 20 °C freezer. Individual coffee bags were defrosted overnight before the day of use.

All coffees were brewed in Curtis G4 Single 1.0 Gal brewers (Model: G4TP2S63A3100, Wilbur Curtis Co., Montebello, CA, USA), with a flat bottomed brew basket and disposable paper filters. This brewer was chosen because it is readily programmable to deliver a wide range of flowrates and brew temperatures. Three brewing temperatures were programmed: 87 °C, 90 °C, and 93 °C. Brewing water was prepared by dissolving 1.16 g CaSO_4_·2H_2_O, 4.97 g MgSO_4_, 3.26 g NaHCO_3_, and 2.57 g KHCO_3_ per litre of deionized water and leaving the solution to equilibrate with ambient CO_2_ until a stable pH near 7 was reached, as specified by industry standards^53^. To control the total extraction time and achieve desired target brews, the brewers were programmed with different “duty cycles” of water pulsing on and off to adjust the average flow rate of water, thus yielding brews at the nine desired locations on the Coffee Brewing Control Chart (cf. Fig. 2). Grind sizes corresponding to the settings 3 (fine), 4 (medium), and 5 (coarse) on a Mahlkönig Guatemala Lab Grinder (Mahlkönig USA, Durham, NC, USA) yielded average particle grind sizes of 770 ± 10 µm, 928 ± 3 µm, and 1115 ± 74 µm; see Frost *et al*.^15^ for detailed grind size distributions. Extraction was controlled between temperatures, so grind, brew ratio of water to coffee, and water pulsing duty cycle were adjusted at different brewing temperatures. The amount of water used to brew was kept consistent at 3075 g of water, while brew ratio was varied by changing the mass of ground coffee. Brew ratios, grind, and duty cycles were chosen through preliminary trials on the study coffee reach the exact target TDS and PE, guided by brew recipes published by Frost et al. on the same brewers^15^. All of the specific brew settings for each of the 27 samples, including brew ratio, grind size, duty cycle, and brew temperature, are listed in Supplementary Table 1.

Brews (with an average mass of 2830.4 g) were collected into insulated thermal carafes (Wilbur Curtis Co. 2.5 L Airpots). Serving began approximately 30 min after brewing, and lasted up to 45 min.

### Panel recruitment and training

Panellists were recruited from the Davis, CA community, giving preference to those who had previously served on descriptive analysis panels, as well as those who had passed a discrimination test screening. Panellists were also required to be regular consumers of black coffee, non-smokers, and not pregnant. All work with human subjects performed here was reviewed and approved by the UC Davis Institutional Review Board (IRB# 1082568) and was performed in accordance with all relevant guidelines and regulations; participants gave informed consent, had freedom to withdraw at any time, and were compensated for all hours. Twelve panellists (four men and eight women) between the ages of 19 and 30 were selected and trained on all samples, three times per week for four weeks. Panellists used the Coffee Taster’s Flavor Wheel for reference and were asked to generate descriptors of the sensory attributes of a training set of coffees^7^. Commonly generated terms were put on an unstructured line scale to create the ballot, generally ordered based on common categories on the Coffee Taster’s Flavor Wheel. On the final ballot agreed upon by consensus of the group, the panel was asked to first smell the coffee and evaluate aromas, before tasting and evaluating the basic tastes, flavour by mouth, and mouthfeel of all attributes listed and categorized accordingly in Table 1. Once comfortable with the ballot, panellists underwent an assessment session in the booths, with identical serving conditions as the final evaluation, after which they were given feedback on their performance and additional, targeted training.

### Descriptive analysis procedure

To compare samples, a generic descriptive analysis that combined elements of the Quantitative Descriptive Analysis and Spectrum methods previously demonstrated successful^13,14,54^ was utilized using Red Jade sensory science software (RedJade, Redwood City, CA, USA). Three aromas, 22 flavours, two mouthfeels, and four basic tastes were evaluated (Table 1). Samples were served in a blind randomized Williams Latin Square block design, with 3-digit blinding codes generated from Red Jade to prevent sample recognition and minimize sensory fatigue. Participants were seated in isolated, positive-pressure, red-lit sensory booths. One to five participants were served per session, each receiving approximately 40 g of each coffee cooled to between 64–66 °C in 200 mL white ceramic mugs before serving. This temperature was chosen to allow for sufficient perception of aroma volatiles before drinking while still affording a comfortably warm consumption temperature. Panellists were instructed first to evaluate the aroma of the hot coffee, and then to evaluate flavour by mouth, taste, and mouthfeel. Panellists recorded attributes via an iPad logged in to Red Jade, with a 15-cm unstructured line scale for each specific sensory attribute from “low” to “high”. Participants tasted one sample at a time and evaluated six samples in a 1-h session, starting with a refresher of the sensory references and then evaluating samples at their own pace, rinsing their mouths with water and unsalted saltine crackers in 3-min breaks between samples. The descriptive analysis took 14 sessions, over which all samples were blind tasted in triplicate.

### Physical measurements of coffee samples

Approximately 150 mL aliquots of coffee from each brew were cooled in sealed containers in an ice bath to room temperature, and the TDS was measured with a VST digital refractometer using one drop of the cooled coffee. Calibration experiments with oven-drying of brewed coffee samples confirmed that the digital refractometer yielded accurate measures of the TDS (see Frost *et al*.^15^ for details). The total delivered mass of the brewed coffee was measured after each brew, and the percent extraction was calculated^3^ as:

1 $$PE= TDS\boldsymbol{ }\times \frac{{M}_{brew}}{{M}_{grounds}}.$$

### Data analysis

#### (M)ANOVA and PCA

Descriptive analysis results were exported from Red Jade, converting positions on the 15-cm line scale into scores from 0 to 100 for each attribute. R version 3.5.1 “Feather Spray” (R Core Team, 2019) was used to perform a five-factor analysis of variance with four-way interactions across participants, replicates, temperature, target PE, and target TDS to determine which attributes were significant, calculated at α = 0.05. F values were adjusted from the original ANOVA by performing a pseudomixed ANOVA to calculate new F ratios and determine which attributes were significant in spite of judge effect^55^. Coffee means for all factors determined to be significant by pseudomixed ANOVA were then compared using Fisher’s Least Significant Difference (LSD) through the Agricolae package in R. Principal Component Analysis plots were produced in R using the FactoMineR package and the ggplot2 package to visually present the selected significant attributes.

#### Response surface methodology

Response surface methodology^56^ was performed using the “rsm” package in R. These calculations were performed with measured TDS and PE values shown in Fig. 2 to account for between-brew index variation when mapping the sensory attributes that the factorial (M)ANOVA omits by analyzing only the target TDS and PE. PE and TDS levels were normalized (Eqs. 2 and 3), and the mean response intensity for each sensory attribute was averaged over all sensory replicates. Regression analysis was performed with a polynomial model shown for the first order fit in Eq. (4), and the second order fit in Eq. (5), as a function for the predicted response $E\left(Y\right)$ with ${\beta }_{0}$ as a constant, and ${\beta }_{1}$**,** ${\beta }_{2}$, ${\beta }_{12}$, ${\beta }_{11}$, and ${\beta }_{22}$ as regression coefficients. ${x}_{1}$ and ${x}_{2}$ are the coded independent brewing control chart variables of PE and TDS, respectively.

2 $${x}_{1}=\frac{PE-20}{4},$$

3 $${x}_{2}=\frac{TDS-1.25}{0.25},$$

4 $$\text{First\;order}: E\left(Y\right)={\beta }_{0}+ {\beta }_{1 }{x}_{1}+{\beta }_{2}{x}_{2},$$

5 $$\text{Second\;order}: E\left(Y\right)={\beta }_{0}+ {\beta }_{1 }{x}_{1}+{\beta }_{2}{x}_{2}+{\beta }_{12}{x}_{1}{x}_{2}+ {\beta }_{11 }{x}_{1}^{2}+{\beta }_{22 }{x}_{2}^{2}.$$

Fisher′s F statistic and lack of fit were evaluated to determine model fit, beginning with first order fit and then applying second order fit only where necessary^56^. If any second order term was significant, the full model was retained.

## Supplementary information

Supplementary Information.
