# Author Correction: Brew temperature, at fixed brew strength and extraction, has little impact on the sensory profile of drip brew coffee

**DOI:** 10.1038/s41598-021-82505-9

**Published:** 2021-01-25

**Authors:** Mackenzie E. Batali, William D. Ristenpart, Jean‑Xavier Guinard

**Affiliations:** 1 Department of Food Science and Technology, University of California, Davis, One Shields Avenue, Davis, CA 95616 USA; 2 Department of Chemical Engineering, University of California, Davis, One Shields Avenue, Davis, CA 95616 USA

Correction to: *Scientific Reports* 10.1038/s41598-020-73341-4, published online 05 October 2020

This article contains a typographical error in the Methods section under subheading ‘Coffee’ where,

"Brewing water was prepared by dissolving 1.16 g CaSO_4_·2H_2_O, 4.97 g MgSO_4_, 3.26 g NaHCO_3_, and 2.57 g KHCO_3_ per litre of deionized water and leaving the solution to equilibrate with ambient CO_2_ until a stable pH near 7 was reached, as specified by industry standards^53^.”

should read:

“Brewing water was prepared by dissolving 1.16 g CaSO_4_·2H_2_O, 4.97 g MgSO_4_, 3.26 g NaHCO_3_, and 2.57 g KHCO_3_ per 100 litres of deionized water and leaving the solution to equilibrate with ambient CO_2_ until a stable pH near 7 was reached, as specified by industry standards^53^.”

