# Supplementary material for: Brew temperature, at fixed brew strength and extraction, has little impact on the sensory profile of drip brew coffee
Source: Sci Rep. 2020 Oct 5;10:16450. doi: 10.1038/s41598-020-73341-4 (PMC7536440; doi:10.1038/s41598-020-73341-4)
Supplement: Supplementary file 1 — Supplementary Information. [file 41598_2020_73341_MOESM1_ESM.pdf]

# Brew temperature, at fixed brew strength and extraction, has little impact on the sensory profile of drip brew coffee

Batali, Mackenzie E.[1], Ristenpart, William D.[2*], Guinard, Jean-Xavier[1]

**Author affiliations**
[1]Department of Food Science and Technology
University of California, Davis
One Shields Avenue
Davis, CA, 95616 USA

[2]Department of Chemical Engineering
University of California, Davis
One Shields Avenue
Davis, CA, 95616 USA

***Corresponding author**
wdristenpart@ucdavis.edu

***Supplementary Figures:***

**S1.** Interaction plots of attributes with significant two-way or three-way interactions.
**S2.** Response surface contour plots of attributes with higher order interactions.
**S3.** Roasting temperature curves.

***Supplementary Tables:***

**S1.** Brewing parameters for all samples, with measured temperature, TDS, and PE values.
**S2.** Table of F-ratios from multivariate analysis of variance.
**S3.** F-ratios for univariate analysis of variance.
**S4.** F-ratios for pseudomixed analysis of variance for Judge/TDS effect and Judge/PE effect.
**S5.** Mean intensities for all measured sensory attributes.

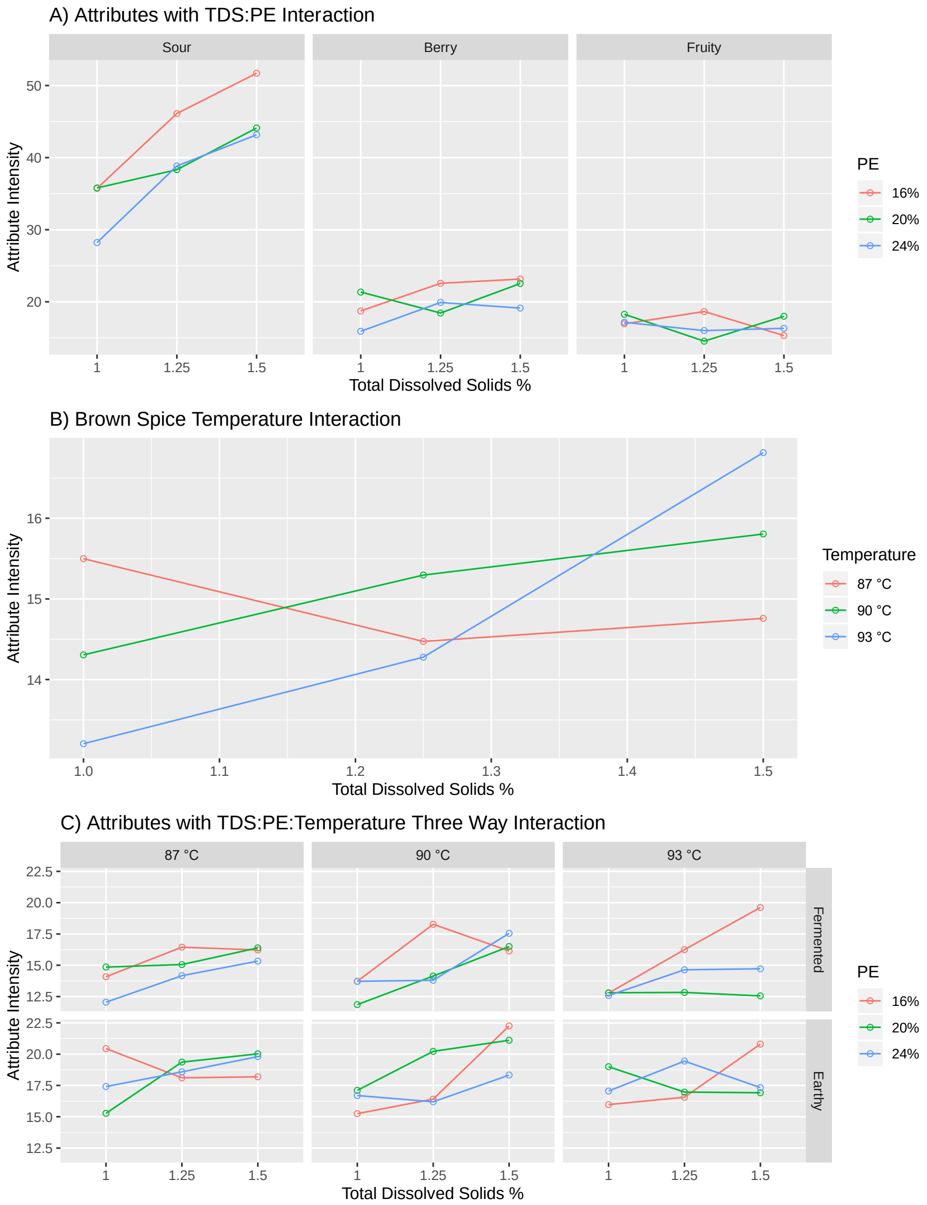


**Supplementary Figure S1.** Interaction plots of attributes with significant two-way (a, b) or three-way (c) interactions.

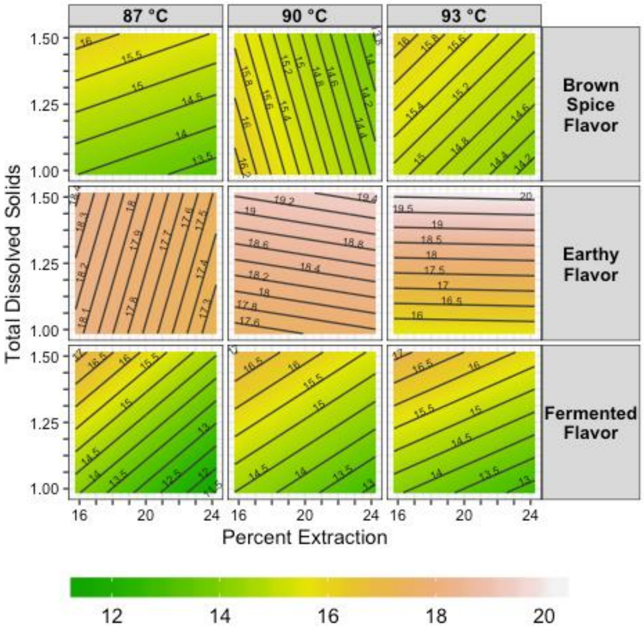


**Supplementary Figure S2.** Response surface methodology contour plots of attributes with Temperature x TDS or Temperature x TDS x PE interactions, to visualize how the interaction effects change the slope of the fit.

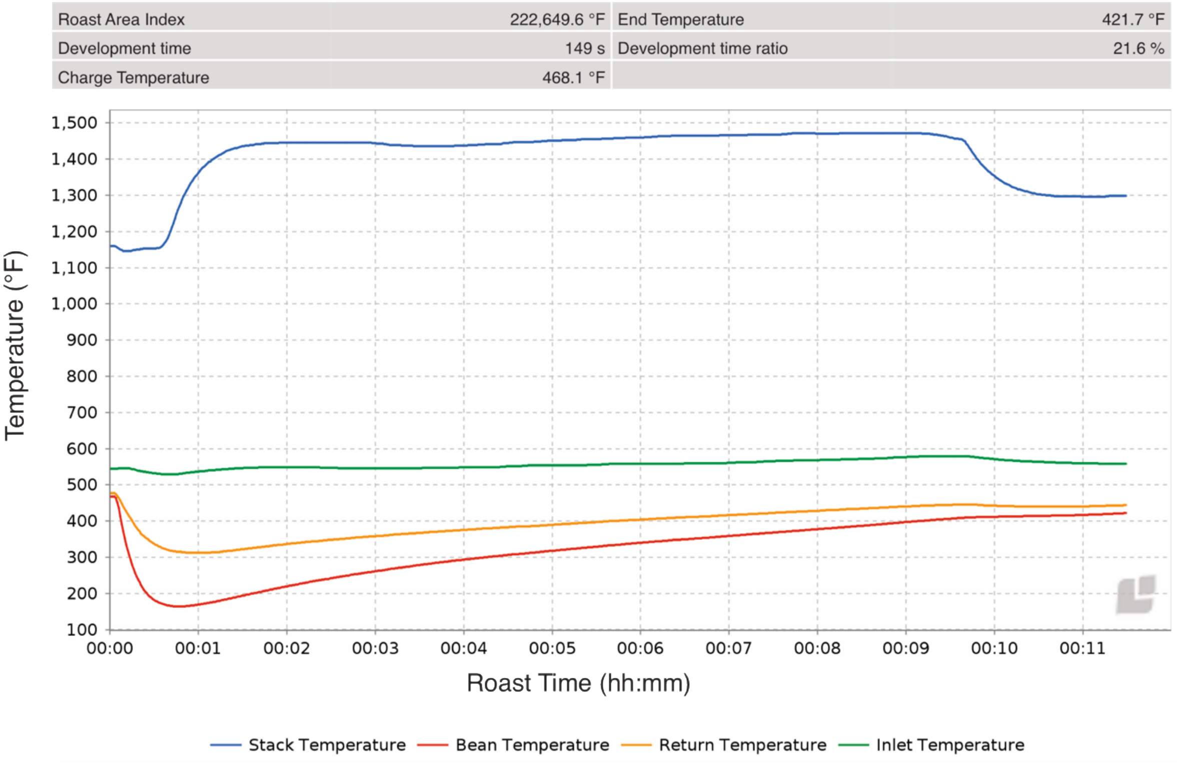


**Supplementary Figure S3.** Roasting temperature curves, as provided by Royal Coffee.

| Target TDS (%) | Target PE (%) | Brew Temp (ºC) | Ground coffee mass (g) | Brew Ratio | Grind Size | Brew Duration (m:ss) | Water on (m:ss) | Water off (m:ss) | Water on/off cycles | Temp post-brew (°C) | Mean TDS (%) | Mean PE (%) |
|---|---|---|---|---|---|---|---|---|---|---|---|---|
| 1.00 | 16 | 87 | 176.1 | 17.5 | 5 | 7:00 | 1:20 | :40 | 2 | 80.4 | 1.05 ± 0.03 | 16.9 ± 0.58 |
| 1.00 | 16 | 90 | 176.1 | 17.5 | 5 | 6:44 | :40 | :16 | 4 | 82.5 | 1.04 ± 0.02 | 17.0 ± 0.38 |
| 1.00 | 16 | 93 | 176.1 | 17.5 | 5 | 6:20 | 1:20 | :20 | 2 | 85.4 | 1.00 ± 0.02 | 16.0 ± 0.23 |
| 1.00 | 20 | 87 | 145.0 | 21.2 | 3 | 6:44 | :40 | :16 | 4 | 80.6 | 1.00 ± 0.02 | 19.7 ± 1.22 |
| 1.00 | 20 | 90 | 145.0 | 21.2 | 3 | 6:44 | :40 | :16 | 4 | 82.2 | 1.03 ± 0.02 | 20.1 ± 1.28 |
| 1.00 | 20 | 93 | 140.3 | 21.9 | 3 | 6:12 | 1:20 | :16 | 2 | 85.0 | 1.02 ± 0.03 | 19.9 ± 1.99 |
| 1.00 | 24 | 87 | 119.0 | 25.8 | 3 | 11:00 | :40 | 1:30 | 4 | 78.4 | 0.99 ± 0.06 | 24.4 ± 1.66 |
| 1.00 | 24 | 90 | 121.1 | 25.4 | 3 | 9:00 | :16 | :20 | 10 | 81.4 | 0.95 ± 0.03 | 22.4 ± 0.95 |
| 1.00 | 24 | 93 | 120.0 | 25.6 | 3 | 11:00 | :40 | 1:30 | 4 | 82.3 | 1.08 ± 0.20 | 24.1 ± 5.34 |
| 1.25 | 16 | 87 | 215.3 | 14.3 | 5 | 7:48 | :20 | :16 | 8 | 79.8 | 1.34 ± 0.03 | 17.6 ± 0.31 |
| 1.25 | 16 | 90 | 215.3 | 14.3 | 5 | 7:00 | :40 | :20 | 4 | 82.0 | 1.28 ± 0.11 | 18.0 ± 2.05 |
| 1.25 | 16 | 93 | 215.3 | 14.3 | 5 | 7:00 | :40 | :20 | 4 | 84.8 | 1.34 ± 0.03 | 17.4 ± 0.45 |
| 1.25 | 20 | 87 | 176.1 | 17.5 | 3 | 7:48 | :20 | :16 | 8 | 80.8 | 1.28 ± 0.02 | 20.5 ± 0.56 |
| 1.25 | 20 | 90 | 176.1 | 17.5 | 3 | 7:48 | :20 | :16 | 8 | 81.7 | 1.29 ± 0.04 | 20.5 ± 1.60 |
| 1.25 | 20 | 93 | 176.1 | 17.5 | 3 | 7:00 | 1:20 | :40 | 2 | 84.6 | 1.33 ± 0.02 | 21.1 ± 0.28 |
| 1.25 | 24 | 87 | 150.0 | 20.5 | 4 | 9:00 | :16 | :20 | 10 | 77.5 | 1.28 ± 0.25 | 24.5 ± 4.74 |
| 1.25 | 24 | 90 | 149.0 | 20.6 | 3 | 11:00 | :40 | 1:30 | 4 | 80.1 | 1.24 ± 0.11 | 23.4 ± 2.19 |
| 1.25 | 24 | 93 | 150.0 | 20.5 | 3 | 9:00 | :16 | :20 | 10 | 83.6 | 1.23 ± 0.04 | 23.1 ± 0.76 |
| 1.50 | 16 | 87 | 252.7 | 12.2 | 5 | 7:28 | :40 | :27 | 4 | 79.4 | 1.53 ± 0.03 | 16.9 ± 0.31 |
| 1.50 | 16 | 90 | 252.7 | 12.2 | 5 | 7:28 | :40 | :27 | 4 | 80.9 | 1.51 ± 0.12 | 17.8 ± 2.07 |
| 1.50 | 16 | 93 | 265.0 | 11.7 | 5 | 6:44 | :40 | :16 | 4 | 83.9 | 1.60 ± 0.11 | 17.8 ± 2.60 |
| 1.50 | 20 | 87 | 207.6 | 14.8 | 3 | 7:28 | :40 | :27 | 4 | 80.2 | 1.45 ± 0.03 | 19.7 ± 0.25 |
| 1.50 | 20 | 90 | 207.6 | 14.8 | 3 | 7:28 | :40 | :27 | 4 | 82.0 | 1.51 ± 0.05 | 20.1 ± 0.71 |
| 1.50 | 20 | 93 | 207.6 | 14.8 | 4 | 8:20 | :40 | :40 | 4 | 83.4 | 1.51 ± 0.06 | 19.4 ± 1.36 |
| 1.50 | 24 | 87 | 176.1 | 17.5 | 3 | 11:00 | :40 | 1:30 | 4 | 78.4 | 1.45 ± 0.06 | 23.2 ± 1.04 |
| 1.50 | 24 | 90 | 176.1 | 17.5 | 3 | 11:00 | :40 | 1:30 | 4 | 80.3 | 1.52 ± 0.16 | 24.5 ± 2.62 |
| 1.50 | 24 | 93 | 176.1 | 17.5 | 3 | 11:00 | :40 | 1:30 | 4 | 82.8 | 1.50 ± 0.06 | 23.8 ± 0.95 |

**Supplementary Table S1.** All brewing parameters for all samples, each sample represented by a unique combination of TDS, PE, and brewing temperature, with average measured TDS and PE and standard deviation. All brews included a 3-minute drip out period at the end of the brew. Grind size refers to setting on a Mahlkönig Guatemala Grinder, with 3 at 770 ± 10 µm, 4 at 928 ± 3 µm, and 5 at 1115 ± 74 µm (reference 15 from main manuscript).

| Factor | df | F-ratio | P-value |
|---|---|---|---|
| *Temperature* | 2 | 1.25 | 0.11 |
| *TDS* | 2 | **5.44** | **<0.001*** ** |
| *PE* | 2 | **2.46** | **<0.001*** ** |
| *Judge* | 11 | **52.69** | **<0.001*** ** |
| *Rep* | 2 | **4.75** | **<0.001*** ** |
| *Judge:Rep* | 22 | **3.01** | **<0.001*** ** |
| *Judge:Temperature* | 22 | 0.96 | 0.72 |
| *Judge:TDS* | 22 | **1.98** | **<0.001*** ** |
| *Judge:PE* | 22 | **1.31** | **<0.001*** ** |
| *Rep:Temperature* | 4 | 0.83 | 0.90 |
| *Rep:TDS* | 4 | 0.83 | 0.89 |
| *Rep:PE* | 4 | 1.12 | 0.19 |
| *Temperature:TDS* | 4 | 1.16 | 0.14 |
| *Temperature:PE* | 4 | 1.07 | 0.30 |
| *TDS:PE* | 4 | **1.56** | **<0.001*** ** |
| *Judge:Rep:Temperature* | 44 | 0.98 | 0.67 |
| *Judge:Rep:TDS* | 44 | **1.16** | **<0.001*** ** |
| *Judge:Rep:PE* | 44 | **1.1** | **0.015* ** |
| *Judge:Temperature:TDS* | 44 | **1.08** | **0.035* ** |
| *Judge:Temperature:PE* | 44 | 1.02 | 0.28 |
| *Judge:TDS:PE* | 44 | **1.10** | **0.0071** ** |
| *Rep:Temperature:TDS* | 8 | 1.09 | 0.18 |
| *Rep:Temperature:PE* | 8 | 1.04 | 0.32 |
| *Rep:TDS:PE* | 8 | 1.15 | 0.068 |
| *Temperature:TDS:PE* | 8 | 1.10 | 0.17 |
| *Judge:Rep:Temperature:TDS* | 88 | **1.09** | **<0.001*** ** |
| *Judge:Rep:Temperature:PE* | 88 | **1.07** | **0.027* ** |
| *Judge:Rep:TDS:PE* | 88 | 1.02 | **0.041* ** |
| *Judge:Temperature:TDS:PE* | 88 | **1.10** | **0.0022** ** |
| *Rep:Temperature:TDS:PE* | 16 | 1.02 | 0. 38 |

**Supplementary Table S2.** Table of F-ratios from Multivariate Analysis of Variance, significance indicated by * (α = 0.05), with corresponding degrees of freedom (df) and p-values.

| Factor | df | Nutty Aroma | Floral Aroma | Green Aroma | Bitter Taste | Sour Taste | Sweet Taste | Salty Taste | Astringent Mouthfeel | Viscous Mouthfeel | Black Tea Flavor | Berry Flavor | Dried Fruit Flavor |
|---|---|---|---|---|---|---|---|---|---|---|---|---|---|
| *Temperature* | 2 | 0.34 | 7.25* | 0.57 | 1.36 | 0.06 | 0.75 | 0.02 | 1.37 | 0.61 | 1.46 | 2.14 | 1.05 |
| *TDS* | 2 | 3.44 | 1.40 | 3.11* | 49.20* | 97.14* | 4.71* | 0.48 | 77.56* | 28.24* | 6.48* | 6.64* | 4.17* |
| *Percent.Extraction* | 2 | 0.051 | 1.80 | 0.89 | 1.53 | 35.04* | 2.39 | 0.82 | 5.74* | 2.41 | 9.80* | 8.40* | 0.16 |
| *Rep* | 2 | 0.61 | 5.12* | 1.22 | 8.03* | 0.44 | 2.90 | 1.63 | 0.22 | 0.65 | 1.98 | 2.48 | 17.81* |
| *Participant.Name* | 11 | 122.65* | 98.42* | 87.64* | 126.4* | 129.05* | 124.28* | 188.39* | 138.77* | 66.65* | 98.37* | 27.91* | 95.33* |
| *Temperature:TDS* | 4 | 0.336 | 0.34 | 0.98 | 0.71 | 2.30 | 0.96 | 1.16 | 0.97 | 0.60 | 0.35 | 1.77 | 1.45 |
| *Temperature:Percent.Extraction* | 4 | 1.28 | 0.06 | 1.92 | 0.26 | 0.96 | 2.28 | 1.07 | 2.23 | 0.77 | 0.98 | 1.19 | 1.17 |
| *Temperature:Rep* | 4 | 0.504 | 0.91 | 1.05 | 0.61 | 0.81 | 0.71 | 0.27 | 1.04 | 0.16 | 0.65 | 0.85 | 1.14 |
| *Temperature:Participant.Name* | 22 | 1.07 | 0.82 | 1.18 | 1.58 | 0.98 | 0.91 | 0.68 | 1.17 | 1.26 | 1.62* | 0.71 | 0.68 |
| *TDS:Percent.Extraction* | 4 | 1.132 | 1.04 | 1.15 | 3.43* | 4.87* | 1.73 | 1.90 | 1.44 | 0.95 | 2.38 | 4.17* | 0.40 |
| *TDS:Rep* | 4 | 0.44 | 0.98 | 2.14 | 2.83* | 0.62 | 0.32 | 1.05* | 1.00 | 1.52 | 1.61 | 0.59 | 0.95 |
| *TDS:Participant.Name* | 22 | 1.744* | 0.60 | 1.69* | 1.92* | 2.78* | 2.11* | 2.11 | 5.56* | 4.74* | 2.30* | 1.13 | 3.67* |
| *Percent.Extraction:Rep* | 4 | 3.14* | 0.66 | 0.90 | 1.00 | 1.39 | 2.19* | 0.76 | 0.87 | 1.60 | 0.74 | 0.36 | 1.64 |
| *Percent.Extraction:Participant.Name* | 22 | 0.960 | 2.83* | 2.13* | 1.39 | 1.75* | 1.53 | 1.16 | 2.21* | 2.08* | 2.32* | 0.85 | 1.61* |
| *Rep:Participant.Name* | 22 | 6.45* | 6.87* | 5.30 | 2.47* | 1.94* | 3.76* | 3.44* | 3.56* | 2.20* | 2.47* | 1.44 | 3.67* |
| *Temperature:TDS:Percent.Extraction* | 8 | 2.26* | 0.85 | 2.25* | 1.18 | 0.74 | 1.11 | 1.36 | 1.00 | 0.48 | 0.87 | 1.05 | 1.43 |
| *Temperature:TDS:Rep* | 8 | 1.88 | 1.86 | 1.08 | 0.68 | 1.31 | 1.14 | 1.92 | 1.07 | 0.73 | 0.88 | 0.61 | 0.94 |
| *Temperature:TDS:Participant.Name* | 44 | 1.27 | 1.49 | 1.18 | 1.02 | 0.70 | 0.95 | 2.28* | 1.11 | 1.04 | 0.77 | 0.88 | 0.99 |
| *Temperature:Percent.Extraction:Rep* | 8 | 1.13 | 1.16 | 1.32 | 0.68 | 0.84 | 1.50 | 0.89 | 1.26 | 1.41 | 0.84 | 1.85 | 0.39 |
| *Temperature:Percent.Extraction:Participant.Name* | 44 | 0.97 | 0.91 | 1.07 | 1.15 | 1.09 | 0.96 | 1.13 | 0.91 | 0.69 | 0.95 | 1.31 | 1.22 |
| *Temperature:Rep:Participant.Name* | 44 | 0.90 | 0.70 | 0.99 | 1.20 | 0.97 | 0.79 | 0.66 | 1.09 | 0.85 | 0.74 | 1.11 | 0.99 |
| *TDS:Percent.Extraction:Rep* | 8 | 0.85 | 1.00 | 1.45 | 1.49 | 1.03 | 1.14 | 1.27 | 1.42 | 0.89 | 0.58 | 1.18 | 0.77 |
| *TDS:Percent.Extraction:Participant.Name* | 44 | 1.26 | 0.95 | 1.15 | 0.93 | 0.99 | 0.85 | 1.32 | 1.19 | 0.69 | 1.49* | 1.69* | 1.05 |
| *TDS:Rep:Participant.Name* | 44 | 1.68* | 1.44 | 1.12 | 1.22 | 0.83 | 1.33* | 1.37 | 1.23 | 1.05 | 0.93 | 1.17 | 1.14 |
| *Percent.Extraction:Rep:Participant.Name* | 44 | 1.01 | 1.36 | 1.02 | 1.06 | 1.43* | 1.65* | 0.68 | 1.21 | 0.84 | 1.18 | 1.11 | 1.04 |
| *Temperature:TDS:Percent.Extraction:Rep* | 16 | 0.73 | 0.88 | 1.30 | 0.98 | 1.90* | 1.11 | 0.71 | 0.74 | 1.47 | 0.61 | 1.78 | 1.32 |
| *Temperature:TDS:Percent.Extraction:Participant.Name* | 88 | 1.35* | 1.11 | 1.02 | 1.60* | 1.09 | 0.98 | 1.19 | 1.18 | 1.18 | 1.36* | 1.10 | 1.26 |
| *Temperature:TDS:Rep:Participant.Name* | 88 | 1.30 | 1.84* | 1.26 | 0.95 | 1.14 | 0.79 | 1.17 | 1.31 | 0.94 | 1.04 | 1.32 | 1.01 |
| *Temperature:Percent.Extraction:Rep:Participant.Name* | 88 | 1.05 | 1.16 | 1.24 | 1.27 | 0.76 | 1.13 | 0.91 | 1.48* | 1.01 | 0.96 | 0.85 | 1.02 |
| *TDS:Percent.Extraction:Rep:Participant.Name* | 88 | 1.21 | 1.11 | 1.01 | 1.22 | 1.04 | 1.05 | 0.87 | 1.42* | 1.14 | 0.83 | 1.16 | 1.04 |

**Supplementary Table S3.** F-ratios for univariate analysis of variance, significance indicated by * (α = 0.05).

| Factor: | df | Fruity Flavor | Citrus Flavor | Fermented Flavor | Fresh Green Flavor | Dark Green Flavor | Woody Flavor | Papery/Musty Flavor | Rubber Flavor | Earthy Flavor | Ashy Flavor |
|---|---|---|---|---|---|---|---|---|---|---|---|
| *Temperature* | 2 | 0.15 | 0.74 | 1.20 | 0.38 | 0.60 | 0.21 | 1.51 | 0.12 | 0.82 | 1.42 |
| *TDS* | 2 | 1.03 | 70.30* | 15.63* | 1.60 | 19.38* | 4.30* | 4.24* | 16.94* | 6.86* | 32.71* |
| *Percent.Extraction* | 2 | 0.22 | 30.58* | 7.17* | 1.02 | 0.94 | 1.94 | 3.23 | 0.91 | 0.43 | 2.05 |
| *Rep* | 2 | 2.03 | 0.34 | 2.25 | 3.19* | 12.34* | 1.22 | 9.88* | 0.19 | 1.50 | 12.35* |
| *Participant.Name* | 11 | 50.21* | 112.79* | 100.17* | 80.39* | 116.59* | 61.72* | 98.94* | 98.88* | 140.1*4 | 67.63* |
| *Temperature:TDS* | 4 | 0.33 | 0.38 | 0.25 | 1.70 | 2.15 | 0.59 | 1.00 | 0.86 | 1.39 | 1.47 |
| *Temperature:Percent.Extraction* | 4 | 0.36 | 0.63 | 2.14 | 1.54 | 1.03 | 0.76 | 1.15 | 0.61 | 1.18 | 0.98 |
| *Temperature:Rep* | 4 | 1.37 | 0.34 | 0.34 | 0.68 | 0.36 | 0.78 | 0.06 | 1.26 | 0.75 | 1.05 |
| *Temperature:Participant.Name* | 22 | 0.75 | 0.84 | 0.78 | 0.95 | 0.57 | 0.18 | 1.17 | 0.61 | 1.46 | 0.73 |
| *TDS:Percent.Extraction* | 4 | 3.38* | 1.56 | 1.24 | 0.55 | 0.14 | 0.46 | 1.05 | 0.68 | 1.31 | 0.77 |
| *TDS:Rep* | 4 | 0.08 | 0.32 | 1.87 | 0.11 | 0.03 | 0.31 | 0.23 | 0.26 | 0.88 | 0.26 |
| *TDS:Participant.Name* | 22 | 0.96 | 2.14* | 1.63* | 1.39 | 2.87* | 2.56* | 1.93* | 4.47* | 2.40* | 5.62* |
| *Percent.Extraction:Rep* | 4 | 0.37 | 0.58 | 0.78 | 0.11 | 0.21 | 0.31 | 0.44 | 1.01 | 0.76 | 1.57 |
| *Percent.Extraction:Participant.Name* | 22 | 0.59 | 1.56 | 1.20 | 0.51 | 0.89 | 0.44 | 0.86 | 0.51 | 1.22 | 1.93* |
| *Rep:Participant.Name* | 22 | 1.00 | 2.88* | 5.52* | 3.29* | 1.34 | 2.12* | 4.56* | 1.41 | 6.12* | 4.72* |
| *Temperature:TDS:Percent.Extraction* | 8 | 1.80 | 0.65 | 1.88* | 0.95 | 1.18 | 0.30 | 0.97 | 1.64 | 2.60* | 0.79 |
| *Temperature:TDS:Rep* | 8 | 0.75 | 0.36 | 0.82 | 1.29 | 2.34* | 0.86 | 0.78 | 0.88 | 0.93 | 0.60 |
| *Temperature:TDS:Participant.Name* | 44 | 0.62 | 1.06 | 0.69 | 0.85 | 1.41 | 1.05 | 1.21 | 0.86 | 1.42* | 0.56 |
| *Temperature:Percent.Extraction:Rep* | 8 | 0.42 | 0.97 | 0.89 | 0.55 | 1.15 | 1.07 | 2.06 | 0.83 | 0.50 | 0.61 |
| *Temperature:Percent.Extraction:Participant.Name* | 44 | 0.81 | 1.19 | 1.52* | 0.95 | 0.82 | 0.91 | 1.25 | 1.00 | 0.90 | 1.14 |
| *Temperature:Rep:Participant.Name* | 44 | 0.63 | 1.06 | 1.02 | 0.62 | 0.82 | 0.87 | 0.79 | 0.62 | 1.35 | 0.85 |
| *TDS:Percent.Extraction:Rep* | 8 | 0.86 | 0.52 | 0.65 | 0.81 | 1.37 | 0.30 | 0.12 | 1.06 | 0.49 | 0.89 |
| *TDS:Percent.Extraction:Participant.Name* | 44 | 0.65 | 1.04 | 0.70 | 0.63 | 1.33 | 0.82 | 1.53 | 0.77 | 1.74* | 0.99 |
| *TDS:Rep:Participant.Name* | 44 | 1.13 | 0.82 | 0.98 | 0.88 | 1.20 | 0.85 | 1.99* | 0.96 | 0.91 | 1.16 |
| *Percent.Extraction:Rep:Participant.Name* | 44 | 0.70 | 1.00 | 0.66 | 1.06 | 1.07 | 0.64 | 1.00 | 1.36 | 1.28 | 1.50* |
| *Temperature:TDS:Percent.Extraction:Rep* | 16 | 0.93 | 1.49 | 0.83 | 0.91 | 0.84 | 0.79 | 1.62 | 0.97 | 0.89 | 0.45 |
| *Temperature:TDS:Percent.Extraction:Participant.Name* | 88 | 0.57 | 1.04 | 1.06 | 0.63 | 1.02 | 0.73 | 1.36 | 1.04 | 1.44* | 1.03 |
| *Temperature:TDS:Rep:Participant.Name* | 88 | 0.96 | 0.95 | 1.85* | 0.79 | 0.79 | 0.77 | 0.78 | 1.20 | 1.41* | 1.01 |
| *Temperature:Percent.Extraction:Rep:Participant.Name* | 88 | 0.93 | 1.03 | 1.06 | 0.80 | 1.04 | 0.63 | 0.95 | 0.86 | 1.09 | 0.77 |
| *TDS:Percent.Extraction:Rep:Participant.Name* | 88 | 0.74 | 1.05 | 1.10 | 0.86 | 1.01 | 0.73 | 1.12 | 0.77 | 0.85 | 0.95 |

**Supplementary Table S3, continued.** F-ratios for univariate analysis of variance, significance indicated by * (α = 0.05).

| Factor: | df | Smoky Flavor | Brown Roast Flavor | Cereal Flavor | Brothy Flavor | Brown Spice Flavor | Nutty Flavor | Dark Chocolate Flavor | Brown Sugar Flavor | Molasses Flavor |
|---|---|---|---|---|---|---|---|---|---|---|
| *Temperature* | 2 | 1.18 | 1.99 | 0.15 | 0.05 | 0.29 | 4.24* | 1.06 | 0.09 | 2.08 |
| *TDS* | 2 | 12.78* | 23.52* | 2.67 | 0.15 | 4.79* | 1.23 | 12.09* | 4.80* | 8.80* |
| *Percent.Extraction* | 2 | 1.37 | 3.42* | 0.90 | 1.81 | 0.89 | 0.84 | 0.07 | 0.12 | 3.21 |
| *Rep* | 2 | 4.92* | 1.67 | 1.72 | 0.06 | 5.36* | 20.10* | 1.43 | 0.65 | 2.84 |
| *Participant.Name* | 11 | 61.18* | 89.97* | 225.77* | 105.53* | 144.5*1 | 252.51* | 28.50* | 49.26* | 76.55* |
| *Temperature:TDS* | 4 | 1.54 | 2.44 | 1.13 | 0.49 | 3.53* | 0.27 | 1.84 | 0.62 | 0.33 |
| *Temperature:Percent.Extraction* | 4 | 0.64 | 0.41 | 0.91 | 0.19 | 1.28 | 0.34 | 1.60 | 0.63 | 0.83 |
| *Temperature:Rep* | 4 | 0.23 | 0.78 | 0.28 | 0.21 | 1.22 | 0.36 | 0.97 | 0.36 | 0.65 |
| *Temperature:Participant.Name* | 22 | 1.14 | 1.26 | 0.49 | 0.45 | 0.90 | 0.50 | 1.22* | 0.61 | 0.43 |
| *TDS:Percent.Extraction* | 4 | 1.16 | 1.12 | 1.10 | 1.23 | 2.38 | 0.57 | 3.26 | 1.51 | 1.38 |
| *TDS:Rep* | 4 | 1.05 | 0.30 | 0.25 | 0.75 | 2.07 | 0.45 | 0.92 | 0.31 | 1.00 |
| *TDS:Participant.Name* | 22 | 5.44* | 3.53* | 0.80 | 0.94 | 1.21 | 0.98 | 4.04* | 2.01* | 4.14* |
| *Percent.Extraction:Rep* | 4 | 0.41 | 0.47 | 1.72 | 0.46 | 1.55* | 1.22 | 1.25 | 2.19 | 0.48 |
| *Percent.Extraction:Participant.Name* | 22 | 0.92 | 0.83 | 1.19 | 1.02 | 1.65* | 0.77 | 1.19 | 0.61 | 1.67 |
| *Rep:Participant.Name* | 22 | 3.48* | 2.71* | 3.16* | 1.98* | 4.98 | 8.60* | 3.55* | 2.46* | 2.91* |
| *Temperature:TDS:Percent.Extraction* | 8 | 0.95 | 0.73 | 0.28 | 0.71 | 0.89 | 1.20 | 1.23 | 0.74 | 1.25 |
| *Temperature:TDS:Rep* | 8 | 1.14 | 1.57 | 1.00 | 1.18 | 2.19* | 0.37 | 1.09 | 1.35 | 0.65 |
| *Temperature:TDS:Participant.Name* | 44 | 0.93 | 1.24 | 0.72 | 0.69 | 1.29 | 0.78 | 1.07 | 0.63 | 0.98 |
| *Temperature:Percent.Extraction:Rep* | 8 | 0.33 | 2.01 | 0.48 | 0.96 | 0.33 | 0.98 | 1.11 | 0.73 | 0.62 |
| *Temperature:Percent.Extraction:Participant.Name* | 44 | 1.06 | 0.76 | 1.03 | 1.04 | 1.24 | 0.91 | 1.10 | 0.57 | 0.61 |
| *Temperature:Rep:Participant.Name* | 44 | 0.73 | 0.91 | 0.75 | 0.70 | 0.79 | 0.59 | 1.11 | 1.20 | 0.97 |
| *TDS:Percent.Extraction:Rep* | 8 | 0.71 | 2.18* | 0.87 | 1.04 | 0.81 | 1.07 | 1.71 | 2.11 | 1.47 |
| *TDS:Percent.Extraction:Participant.Name* | 44 | 1.19 | 1.24 | 0.86 | 0.88 | 0.61 | 0.81 | 1.85* | 0.91 | 0.65 |
| *TDS:Rep:Participant.Name* | 44 | 1.24 | 1.22 | 0.95 | 1.51 | 1.07 | 0.98 | 2.22* | 0.59 | 1.39 |
| *Percent.Extraction:Rep:Participant.Name* | 44 | 1.33 | 1.60* | 0.83 | 0.58 | 1.55* | 1.03 | 1.87* | 1.00 | 0.65 |
| *Temperature:TDS:Percent.Extraction:Rep* | 16 | 1.56 | 1.54 | 1.08 | 0.37 | 1.14 | 0.86 | 1.26 | 0.98 | 0.93 |
| *Temperature:TDS:Percent.Extraction:Participant.Name* | 88 | 1.49 | 0.91 | 0.79 | 1.12 | 1.19 | 0.67 | 0.94 | 0.68 | 1.41 |
| *Temperature:TDS:Rep:Participant.Name* | 88 | 1.16 | 0.86 | 0.79 | 0.74 | 0.97 | 0.90 | 1.24 | 0.94 | 0.85 |
| *Temperature:Percent.Extraction:Rep:Participant.Name* | 88 | 0.91 | 1.22 | 0.88 | 0.84 | 1.01 | 0.84 | 1.11 | 1.10 | 1.44 |
| *TDS:Percent.Extraction:Rep:Participant.Name* | 88 | 0.95 | 0.90 | 1.07 | 0.78 | 0.81 | 0.68 | 1.18 | 1.10 | 1.54 |

**Supplementary Table S3, continued.** F-ratios for univariate analysis of variance, significance indicated by * (α = 0.05).

| Attribute | Judge/TDS adjusted F-ratio | Judge/PE adjusted F-ratio |
|---|---|---|
| Nutty Aroma | 1.70 | 0.089 |
| Floral Aroma | 2.35 | 0.63 |
| Green/Vegetative Aroma | 1.84 | 0.42 |
| Bitter Taste | **25.65*** | 1.09 |
| Sour Taste | **34.91*** | **20.02*** |
| Sweet Taste | 2.23 | 1.57 |
| Salty Taste | 0.23 | 0.71 |
| Black Tea Flavor | 2.81 | **4.22*** |
| Berry Flavor | **5.87*** | **9.87*** |
| Dried Fruit Flavor | 1.14 | 0.098 |
| Fruity Flavor | 1.07 | 0.37 |
| Citrus Flavor | **32.91*** | 19.62 |
| Fermented Flavor | **9.56*** | **5.99*** |
| Fresh Green Flavor | 1.15 | 2.03 |
| Dark Green Flavor | **6.74*** | 1.05 |
| Woody Flavor | 1.68 | **4.43*** |
| Papery/Musty Flavor | 2.20 | **3.75*** |
| Rubber Flavor | **3.79*** | 1.80 |
| Earthy Flavor | 2.86 | 0.35 |
| Ashy Flavor | **5.81*** | 1.06 |
| Smoky Flavor | 2.34 | 1.5 |
| Brown Roast Flavor | **6.66*** | **4.12*** |
| Cereal Flavor | 3.35 | 0.75 |
| Brothy Flavor | 0.16 | 1.79 |
| Brown Spice Flavor | 3.97* | 0.54 |
| Nutty Flavor | 1.25 | 1.09 |
| Dark Chocolate Flavor | 3.00 | 0.060 |
| Brown Sugar Flavor | 2.39 | 0.20 |
| Molasses Flavor | 2.13 | 1.93 |
| Astringent Mouthfeel | **13.95*** | 2.60 |
| Viscous Mouthfeel | **5.95*** | 1.16 |

**Supplementary Table S4.** F-ratios for pseudomixed analysis of variance for Judge/TDS effect and Judge/PE effect, each with 2 degrees of freedom, significance indicated by * ($\alpha = 0.05$).

| Attribute | Total Dissolved Solids | | | Percent Extraction | | | Brew Temperature | | | Fischer's LSD |
|---|---|---|---|---|---|---|---|---|---|---|
| | 1% | 1.25% | 1.50% | 16% | 20% | 24% | 87 °C | 90 °C | 93 °C | |
| Nutty Aroma | 33.4 | 33.54 | 35.4 | 34.26 | 34.18 | 33.91 | 33.9 | 34.54 | 33.91 | |
| Floral Aroma | 26.52 | 27.96 | 27.12 | 28.12 | 26.9 | 26.57 | 28.24 | 28.05 | 25.31 | |
| Green/Vegetative Aroma | 28.68 | 30.77 | 28.63 | 30.03 | 28.73 | 29.32 | 28.84 | 29.88 | 29.36 | |
| Bitter Taste | **35.26**$^{b}$ | **41.68**$^{a}$ | **43.59**$^{a}$ | 39.29 | 40.56 | 40.67 | 39.41 | 40.26 | 40.85 | 1.74 |
| Sour Taste | **33.25**$^{c}$ | **41.1**$^{b}$ | **46.33**$^{a}$ | **44.52**$^{a}$ | **39.41**$^{b}$ | **36.74**$^{c}$ | 40.04 | 40.33 | 40.3 | 1.86 |
| Sweet Taste | 22.98 | 21.29 | 20.76 | 22.62 | 21.09 | 21.31 | 22.21 | 21.46 | 21.36 | |
| Salty Taste | 13.65 | 13.96 | 14.13 | 13.89 | 14.24 | 13.6 | 13.94 | 13.85 | 13.94 | |
| Black Tea Flavor | 28.09 | 25.63 | 25.26 | **24.24**$^{b}$ | **26.79**$^{a}$ | **27.94**$^{a}$ | 26.88 | 25.5 | 26.59 | 1.67 |
| Berry Flavor | **18.65**$^{b}$ | **20.3**$^{b}$ | **21.6**$^{b}$ | **21.47**$^{a}$ | **20.77**$^{a}$ | **18.31**$^{b}$ | 21.1 | 19.99 | 19.46 | 1.66 |
| Dried Fruit Flavor | 21.8 | 23.56 | 24.2 | 23.45 | 23.13 | 22.98 | 22.98 | 23.89 | 22.69 | |
| Fruity Flavor | 17.45 | 16.39 | 16.54 | 16.97 | 16.93 | 16.49 | 17.03 | 16.6 | 16.74 | |
| Citrus Flavor | **28.25**$^{a}$ | **34.53**$^{b}$ | **39.8**$^{c}$ | **38.41** | **33.17** | **31** | 33.83 | 33.87 | 34.88 | 1.92 |
| Fermented Flavor | **13.16**$^{a}$ | **15.07**$^{b}$ | **16.11**$^{c}$ | **15.95** | **14.11** | **14.29** | 14.31 | 14.96 | 15.08 | 1.06 |
| Fresh Green Flavor | 20.95 | 19.92 | 19.51 | 20.8 | 19.67 | 19.91 | 19.97 | 19.87 | 20.54 | |
| Dark Green Flavor | **22.65**$^{b}$ | **27.26**$^{a}$ | **28.04**$^{a}$ | 26.67 | 25.4 | 25.89 | 25.46 | 26.48 | 26.02 | 1.85 |
| Woody Flavor | 18.21 | 19.84 | 20.76 | 18.73 | 19.61 | 20.47 | 19.36 | 19.53 | 19.91 | |
| Papery/Musty Flavor | 16.89 | 18.76 | 18.45 | 17.07 | 18.24 | 18.79 | 17.36 | 18.23 | 18.51 | |
| Rubber Flavor | **13.71**$^{c}$ | **15.8**$^{b}$ | **17.82**$^{a}$ | 15.91 | 15.25 | 16.17 | 15.81 | 15.59 | 15.93 | 1.39 |
| Earthy Flavor | **17.14**$^{b}$ | **17.98**$^{b}$ | **19.42**$^{a}$ | 18.22 | 18.44 | 17.87 | 17.78 | 18.58 | 18.18 | 1.23 |
| Ashy Flavor | **18.13**$^{c}$ | **22.84**$^{b}$ | **25.24**$^{a}$ | 21.02 | 22.54 | 22.65 | 21.27 | 22.17 | 22.77 | 1.77 |
| Smoky Flavor | 16.5 | 18.52 | 20.09 | 18.9 | 18.47 | 17.74 | 18.04 | 18.07 | 19 | |
| Brown Roast Flavor | **29.52**$^{c}$ | **33.16**$^{b}$ | **35.21**$^{a}$ | **31.37** | **33.14** | **33.38** | 31.66 | 33.04 | 33.19 | 1.7 |
| Cereal Flavor | 21.5 | 21.36 | 19.94 | 20.98 | 21.41 | 20.41 | 21.07 | 20.7 | 21.03 | |
| Brothy Flavor | 15.41 | 15.59 | 15.19 | 16.14 | 15.31 | 14.76 | 15.47 | 15.26 | 15.47 | |
| Brown Spice Flavor | **14.34**$^{b}$ | **14.68**$^{b}$ | **15.79**$^{a}$ | 14.95 | 15.26 | 14.6 | 14.77 | 14.91 | 15.14 | 0.97 |
| Nutty Flavor | 27.52 | 26.89 | 26.4 | 27.47 | 26.59 | 26.76 | **26.07**$^{b}$ | **28.1**$^{a}$ | **26.65**$^{b}$ | 1.42 |
| Dark Chocolate Flavor | 19.72 | 21.68 | 23.46 | 21.46 | 21.67 | 21.73 | 21.06 | 22.16 | 21.64 | |
| Brown Sugar Flavor | 19.48 | 17.34 | 17.17 | 17.84 | 18.23 | 17.93 | 17.8 | 18.12 | 18.08 | |
| Molasses Flavor | 14.22 | 15.17 | 16.8 | 16.12 | 15.51 | 14.56 | 15.3 | 16.07 | 14.82 | |
| Astringent Mouthfeel | **29.88**$^{c}$ | **36.19**$^{b}$ | **40.49**$^{a}$ | 36.88 | 35.7 | 33.99 | 35.16 | 35.07 | 36.34 | 1.69 |
| Viscous Mouthfeel | **29.28**$^{b}$ | **35**$^{a}$ | **35.6**$^{a}$ | 32.98 | 34.43 | 32.47 | 32.75 | 33.77 | 33.35 | 1.83 |

**Supplementary Table S5.** Mean attribute intensities for all measured variables with Fischer's LSD significance codes, shaded by aromas, basic tastes, flavors, and mouthfeels, in order.
